# Supplementary material for: How crystallization additives govern halide perovskite grain growth
Source: Nat Commun. 2025 Nov 10;16:9894. doi: 10.1038/s41467-025-65484-7 (PMC12603034; doi:10.1038/s41467-025-65484-7)
Supplement: Supplementary file 1 — Supplementary Information [file 41467_2025_65484_MOESM1_ESM.pdf]

Supporting Information for

How Crystallization Additives Govern Halide Perovskite Grain Growth

Timo Maschwitz[1,2 †], Lena Merten[3,4 †], Feray Ünlü[5,6], Martin Majewski[7], Fatemeh Haddadi Barzoki[8], Zijin Wu[9,10], Seren Dilara Öz[11], Cedric Kreusel[1,2], Manuel Theisen[1,2], Pang Wang[1,2], Maximilian Schiffer[1,2], Gianluca Boccarella[1,2], Gregor Marioth[1,2], Henrik Weidner[1,2], Sarah Schultheis[1,2], Tim Schieferstein[1,2], Dawid Gidaszewski[12], Zavkiddin Julliev[13], Ekaterina Kneschaurek[3], Valentin Munteanu[3], Ivan Zaluzhnyy[3], Florian Bertram[14], Anaël Jaffrès[15], Junjie He[1,16], Nigmat Ashurov[13], Martin Stolterfoht[17], Christian M. Wolff[15], Eva Unger[6], Selina Olthof[11], Geert Brocks[9,10,18], Shuxia Tao[9,10], Helen Grüninger[8], Olivier J.J. Ronsin[7], Jens Harting[7], Andreas F. Kotthaus*[19], Stefan F. Kirsch[19], Sanjay Mathur[5], Alexander Hinderhofer*[3,20], Frank Schreiber[3,20], Thomas Riedl*[1,2], Kai O. Brinkmann*[1,2]
[†] contributed equally

* Kai O. Brinkmann: brinkmann@uni-wuppertal.de
* Thomas Riedl: t.riedl@uni-wuppertal.de
* Alexander Hinderhofer: alexander.hinderhofer@uni-tuebingen.de
* Andreas Kotthaus: kotthaus@uni-wuppertal.de

[1] Chair of Electronic Devices, University of Wuppertal, Rainer-Gruenter-Straße 21, 42119 Wuppertal
[2] Wuppertal Center of Smart Materials and Systems, University of Wuppertal, Rainer-Gruenter-Straße 21, 42119 Wuppertal, Germany
[3] Institute of Applied Physics, University of Tübingen, Auf der Morgenstelle 10, 72076 Tübingen, Germany
[4] Division of Physical Chemistry, Lund University, Naturvetarvägen 14, 22100 Lund, Sweden
[5] Department of Chemistry, University of Cologne, Greinstraße 4-6, 50939 Cologne, Germany
[6] HySPRINT Innovation Lab, Helmholtz-Zentrum Berlin für Materialen und Energie GmbH, Kekuléstraße 5, 12489 Berlin, Germany
[7] Helmholtz Institute Erlangen-Nürnberg for Renewable Energy (HIERN), Forschungszentrum Jülich, Fürther Straße 248, 90429 Nürnberg, Germany
[8] Inorganic Chemistry III and Bavarian Center for Battery Technology (BayBatt), University of Bayreuth, Universitätsstr. 30, 95447 Bayreuth, Germany

[9] Materials Simulation & Modelling, Department of Applied Physics and Science Education, Eindhoven University of Technology, 5600 MB, Eindhoven, The Netherlands
[10] Center for Computational Energy Research, Department of Applied Physics and Science Education, Eindhoven University of Technology, 5600 MB Eindhoven, The Netherlands
[11] Chair for Material and Surface Analysis, University of Wuppertal, Rainer-Gruenter-Straße 21, 42119 Wuppertal, Germany
[12] Eindhoven Institute for Renewable Energy Systems (EIRES) Eindhoven University of Technology 5600 MB Eindhoven, The Netherlands
[13] Institute of Polymer Chemistry and Physics, Academy of Science of the Republic of Uzbekistan, 100128, Tashkent, Uzbekistan
[14] Deutsches Elektronen-Synchrotron DESY, 22607 Hamburg, Germany
[15] Institute of Electrical and Microengineering (IEM), Ecole Polytechnique Fédérale de Lausanne (EPFL), Photovoltaics and Thin-Film Electronics Laboratory, 2002 Neuchâtel, Switzerland
[16] Department of Science and Technology, Yunnan Agricultural University, Kunming 650201, China
[17] Electronic Engineering Department, The Chinese University of Hong Kong, Hong Kong, SAR China
[18] Computational Chemical Physics, Faculty of Science and Technology and MESA+ Institute for Nanotechnology, University of Twente, 7500 AE Enschede, The Netherlands
[19] Department of Organic Chemistry, Bergische Universität Wuppertal, Gaußstraße 20, 42119 Wuppertal, Germany
[20] LISA+, University of Tübingen, Auf der Morgenstelle 15, 72076 Tübingen, Germany

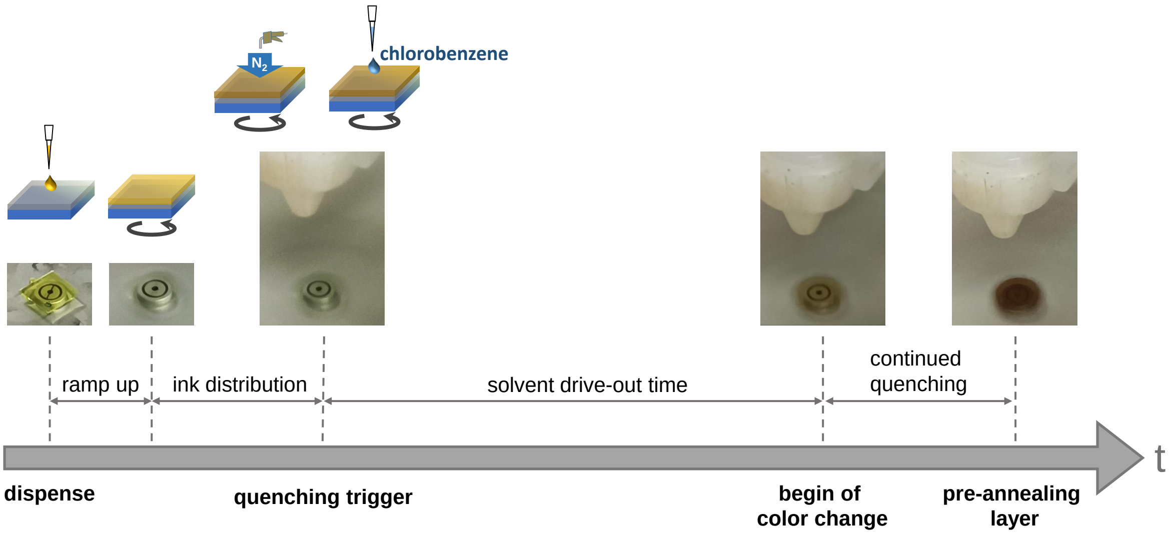

Figure S1: Schematic illustration how the "solvent drive out time" was determined, as the timespan between the trigger and the color change on the example of a gas-quenching process. We want to note, that for slower processes, the color change was a gradual process and determination of the solvent drive-out time is therefore subject to some uncertainty. We recorded the timespan until the first sign of an increased film absorption was visible with the eye, as shown in the picture over "color change" compared to the color observable over "quenching trigger". Consequently, the timespans stated should be understood as an estimation.

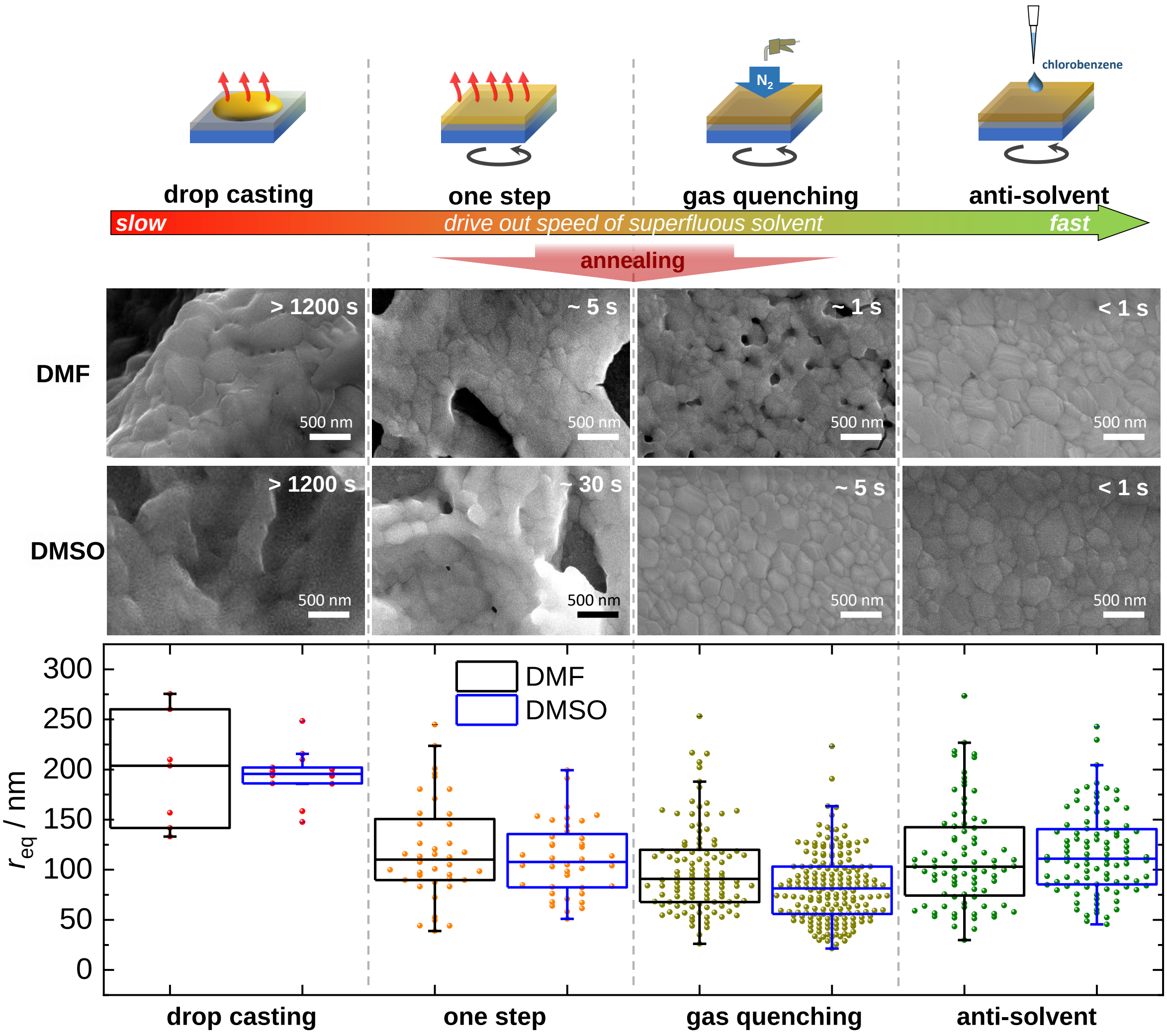

Figure S2: $MAPbI_3$ grain formation in dependency of the solvent drive out speed (see Fig. S1). The drive-out speed was altered by using drop casting, one step, gas-quenching and anti-solvent perovskite deposition techniques. The top row shows SEM images of the films produced from DMF and DMSO-based precursor solutions. The bottom row shows the equivalent radii (r_{eq}) of the perovskite grains evaluated from SEM images in accordance to Supplementary Note 1.

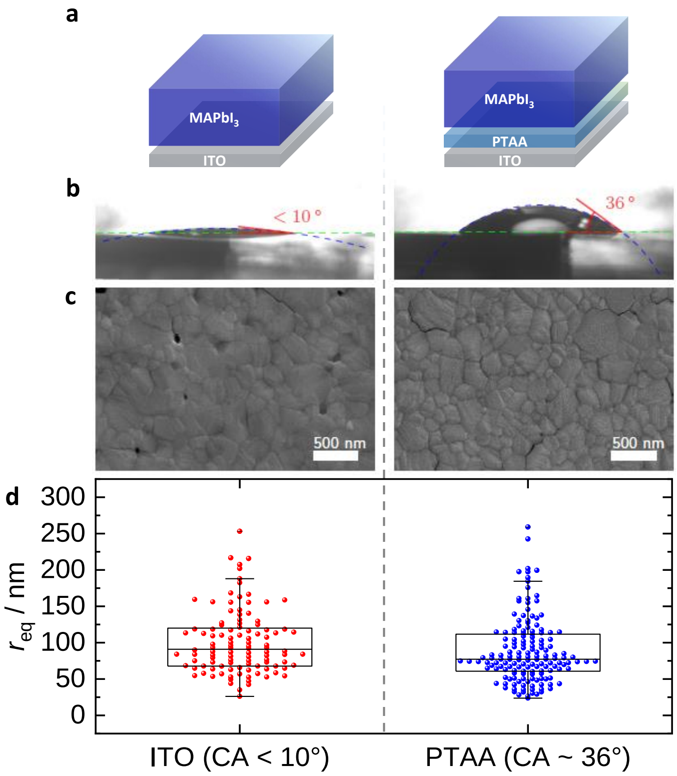

Figure S3: Perovskite formation on hydrophilic (left) and hydrophobic (right) substrate. **a,** schematic layer stack **b,** DMF contact angle with the respective ITO or PTAA-ITO substrate **c,** top-view SEM images and **d,** equivalent radii of grains derived by watershed algorithm from the SEM images.

Supplementary Note 1

For the evaluation of the grain sizes, SEM images were taken perpendicular to the surface of the substrate with a secondary electron detector. To have a sufficient conductance of the sample, ITO-coated glass substrates were used for all SEM samples. The images were then processed with the water shed algorithm of Gwyddion to achieve areas which are separated by lines. These areas were then used to evaluate the grain size with the Gwyddion software; for each area a radius was calculated which corresponds to a circle with the same area—so-called equivalent radius.

For images that do not show enough contrast or have other imperfections, the grain boundaries were drawn in by hand in a secondary layer in a graphics program. The secondary layer was exported as a raster graphic and processed in Gwyddion the same way as the result from the water shed algorithm. Please note, that due to the irregular morphology, also the incident angle of the electron beam cannot be guaranteed to be perpendicular to the surface for perovskites deposited by the drop-casting method. Therefore, some projection related misinterpretation cannot be entirely excluded.

a

b

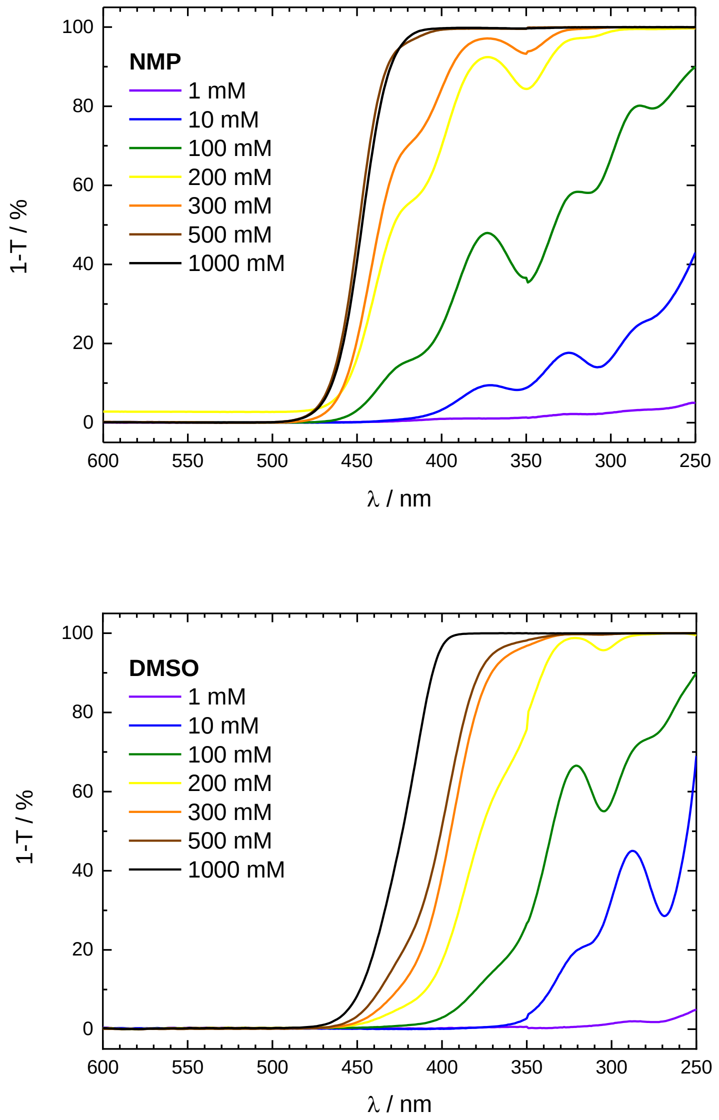

Figure S4: UV-Vis spectra of $MAPbI_3$ precursor inks with different concentrations in **a,** NMP and **b,** DMSO

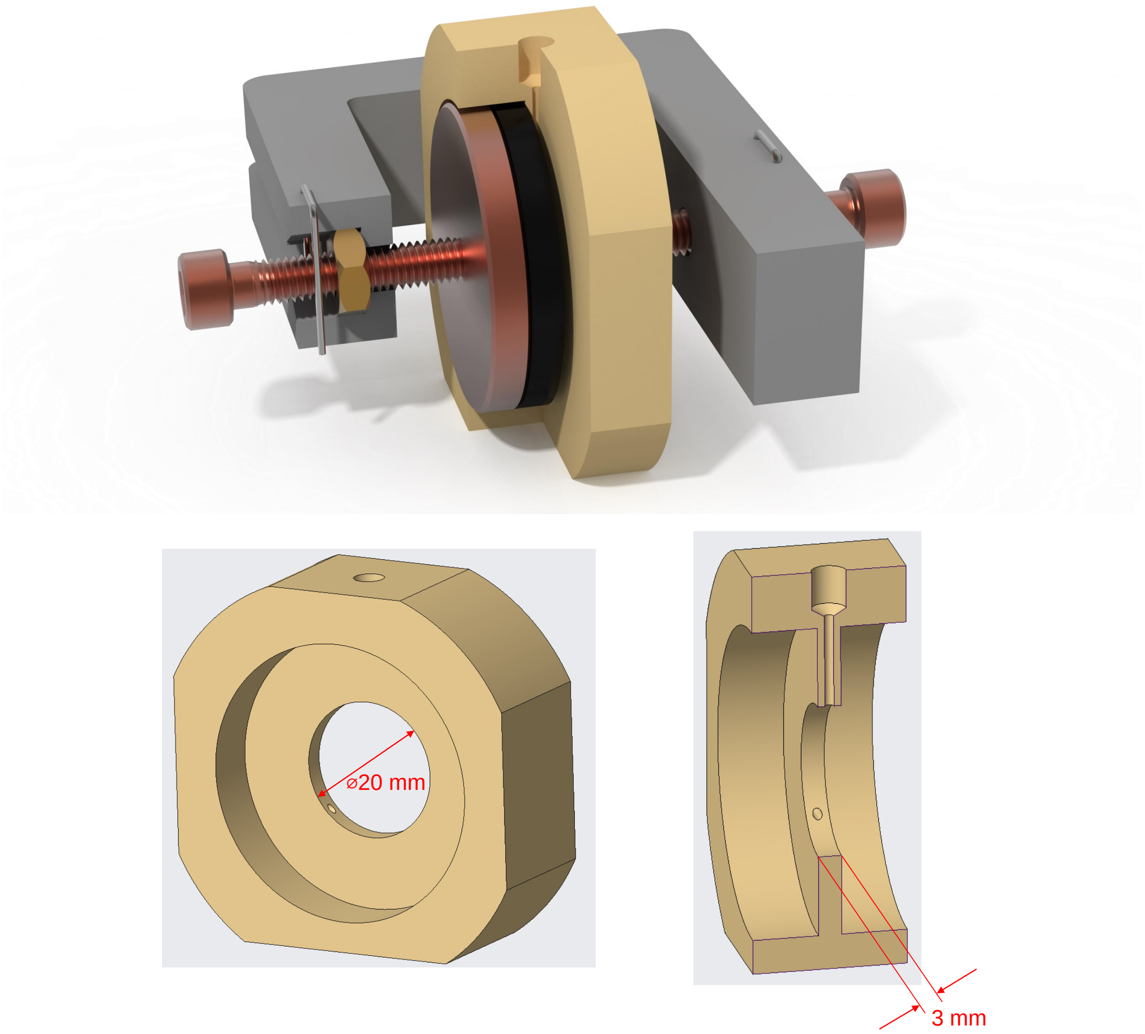

Figure S5: Sketch of the measurement cell used for the conductance measurement.

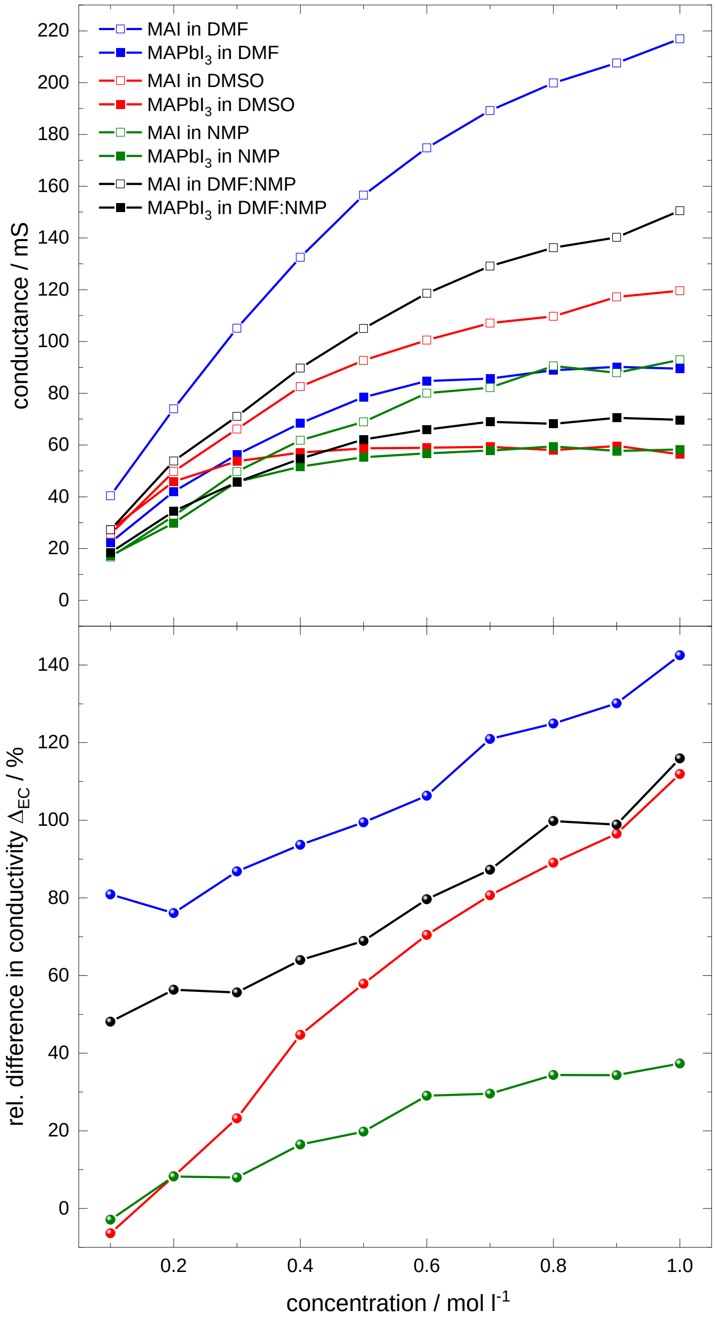

Figure S6: Electrical conductance (EC) of $MAPbI_3$ (top) and relative difference of conductance between the dissociated MAI and the colloidal $MAPbI_3$ ink, referenced by $MAPbI_3$, calculated as $\Delta_{EC} = \frac{G_{MAI} - G_{MAPbI_3}}{G_{MAPbI_3}}$, (bottom) of NMP and a 7:3 DMF:NMP mixture.

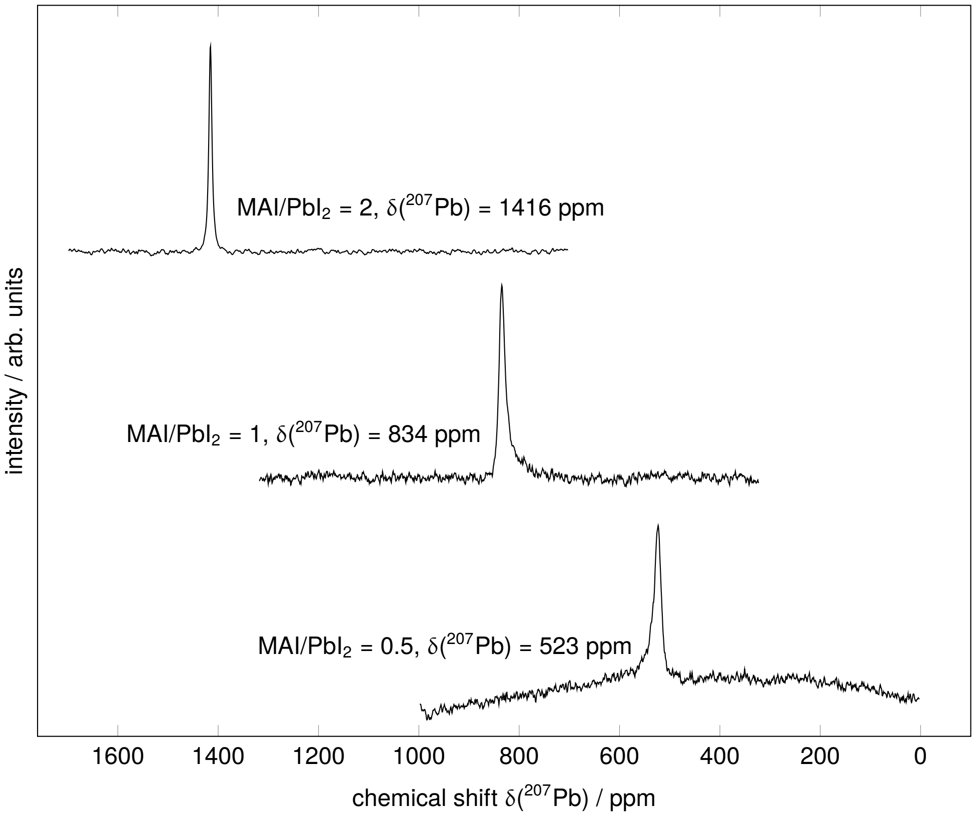

Figure S7: ^{207}Pb-NMR chemical shifts of the Pb^{2+} nuclei in solutions containing either excessive PbI_2 or MAI. It is observable how a change in the Pb^{2+}-to-iodide or the Pb^{2+}-to-MA ratio in the ink can impact on the measurement. Additives like $Pb(SCN)_2$ or MACl would similarly alter the Pb^{2+}-to-iodide or the Pb^{2+}-to-MA ratio and are therefore unsuitable examples for our case study, as they might create false positives.

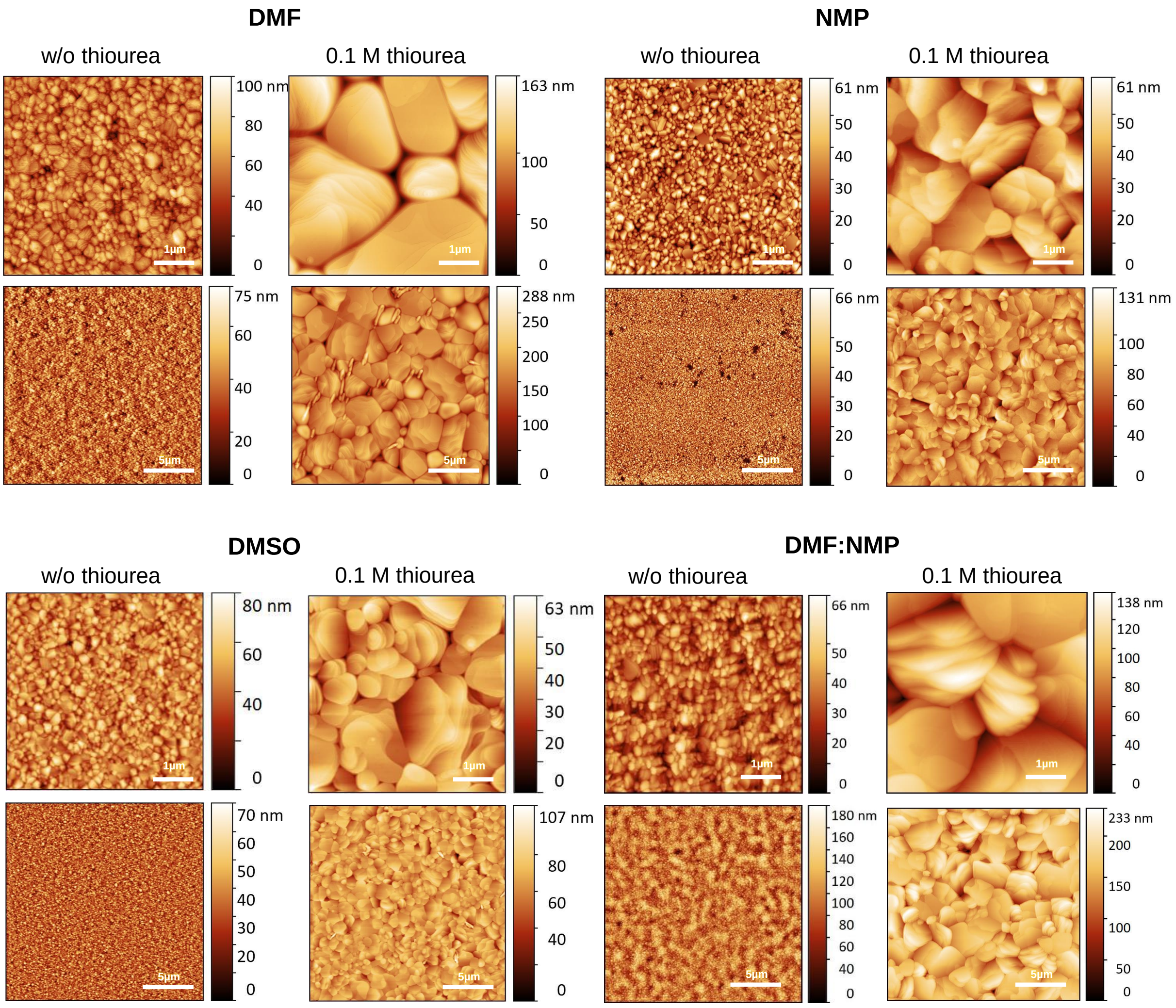

Figure S8: AFM topography images of $MAPbI_3$ layers deposited on ITO with and w/o 0.1 M thiourea additive in pure solvents DMF, NMP and DMSO and DMF:NMP.

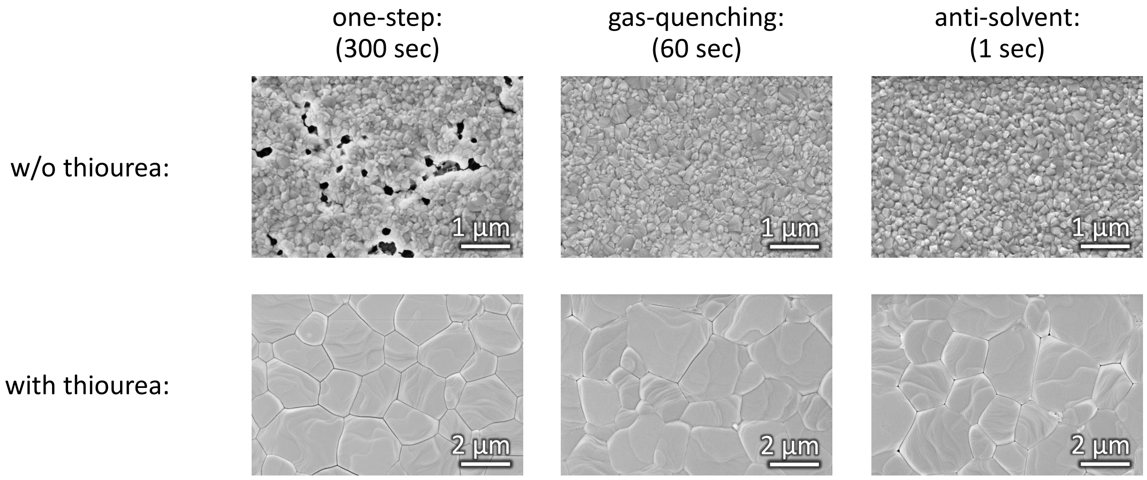

Figure S9: Impact of 0.1 M of thiourea additive on $MAPbI_3$ grain size in dependence of the deposition technique. The SEM data shows the independence of the effect from the solvent drive-out speed (compare Fig. S2).

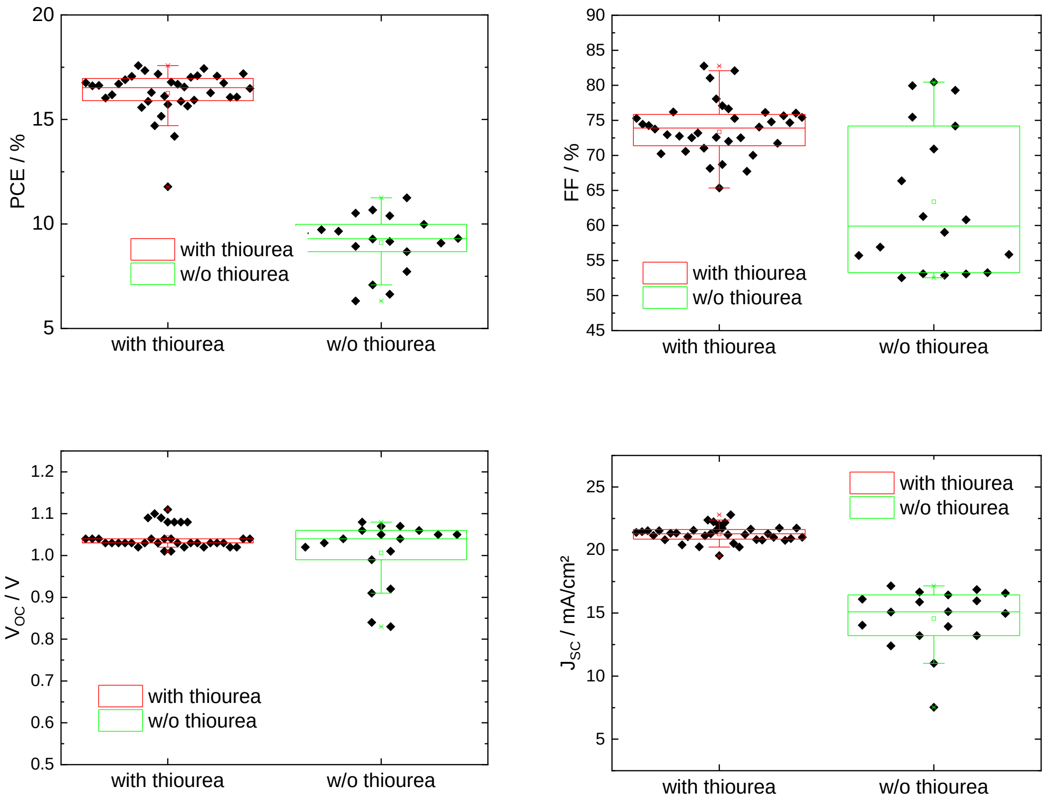

Figure S10: Statistics of solar cells employing $MAPbI_3$ with and w/o 0.1 M thiourea additive. Upper and lower box ranges depict interquartile range (25 % – 75 %) with a median line in between. The whiskers show the ultimate data point inside yet another 1.5 interquartile range to identify outliers. Please note, that data shown w/o thiourea represents the best recorded J-V characteristic and not the stabilized PCE and is therefore likely even lower in the steady state. Typical stabilized PCE data for both cases are shown in Fig. S11

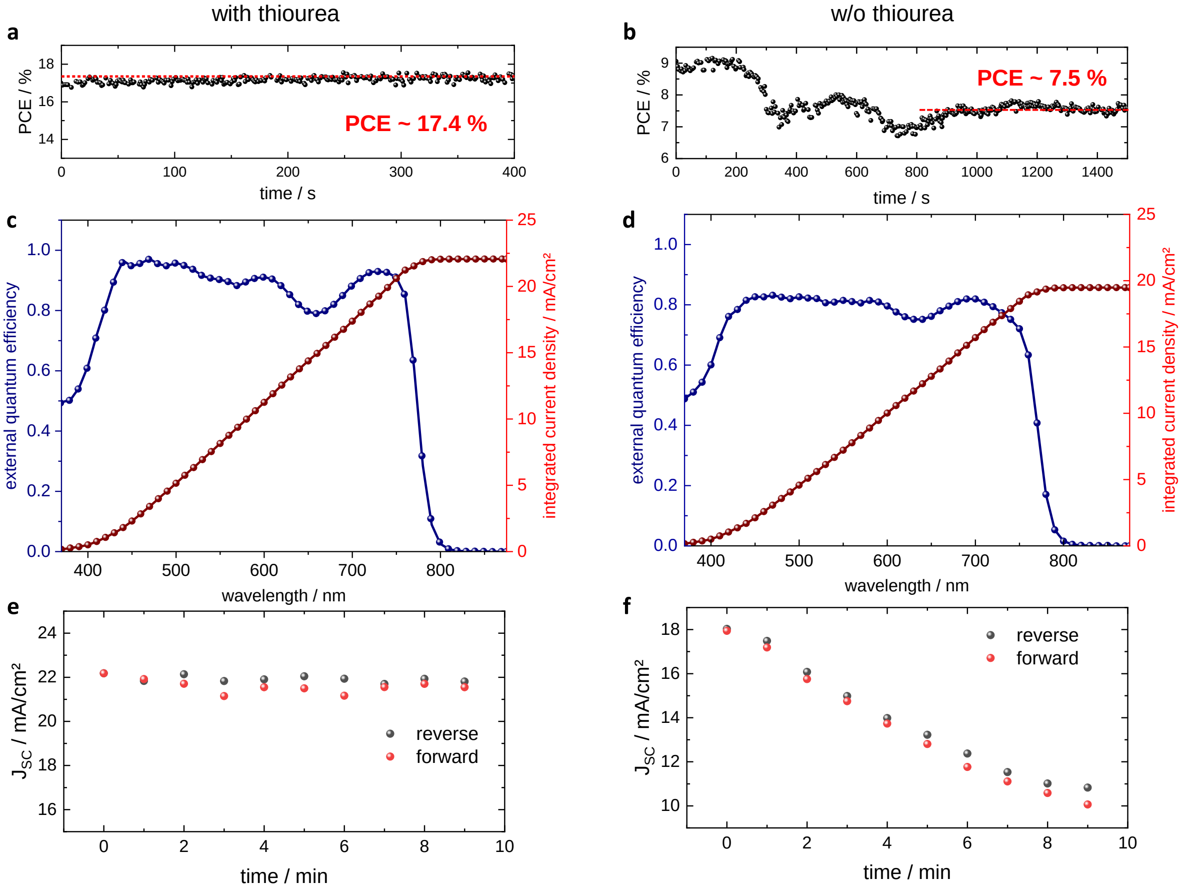

Figure S11: a, b, stabilized power conversion efficiencies, **c, d,** EQE before light soaking and **e, f,** the development of the short circuit current density during light soaking, of $MAPbI_3$ solar cells employing either 0.1 м thiourea (**a**, **c**, **e**) or were processed without thiourea additive (**b**, **d**, **f**).

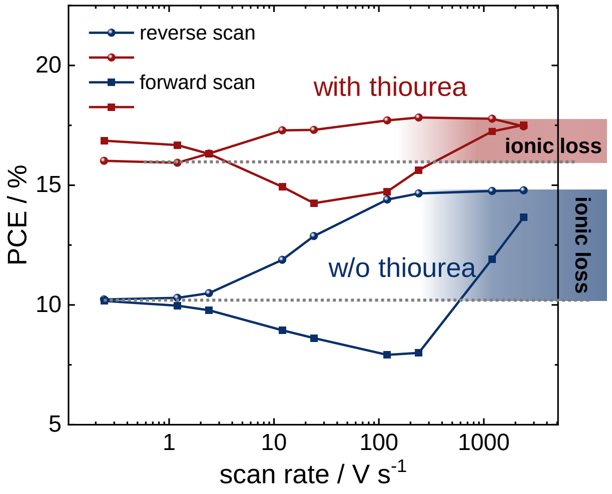

Figure S12: Fast hysteresis measurement of $MAPbI_3$ solar cells both with and w/o thiourea additive showcasing the losses due to mobile ions in both devices. The measurement and analysis have been conducted in analogy to our previous work.[1] Note that the measurements have been taken after light soaking, because a steady state is required for meaningful results.

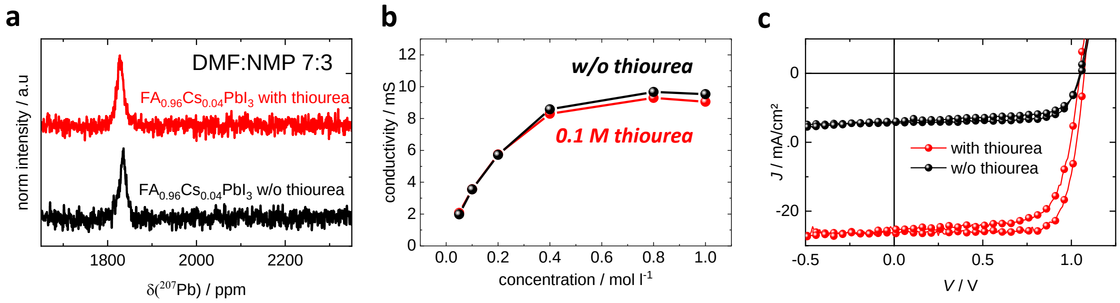

Figure S13: a, ^{207}Pb-NMR spectra and **b,** conductance measurements of $FA_{0.94}Cs_{0.06}PbI_3$ in DMF:NMP solvent mixture with and w/o 0.1 M of thiourea additive. **c,** J-V characteristics of respective solar cells.

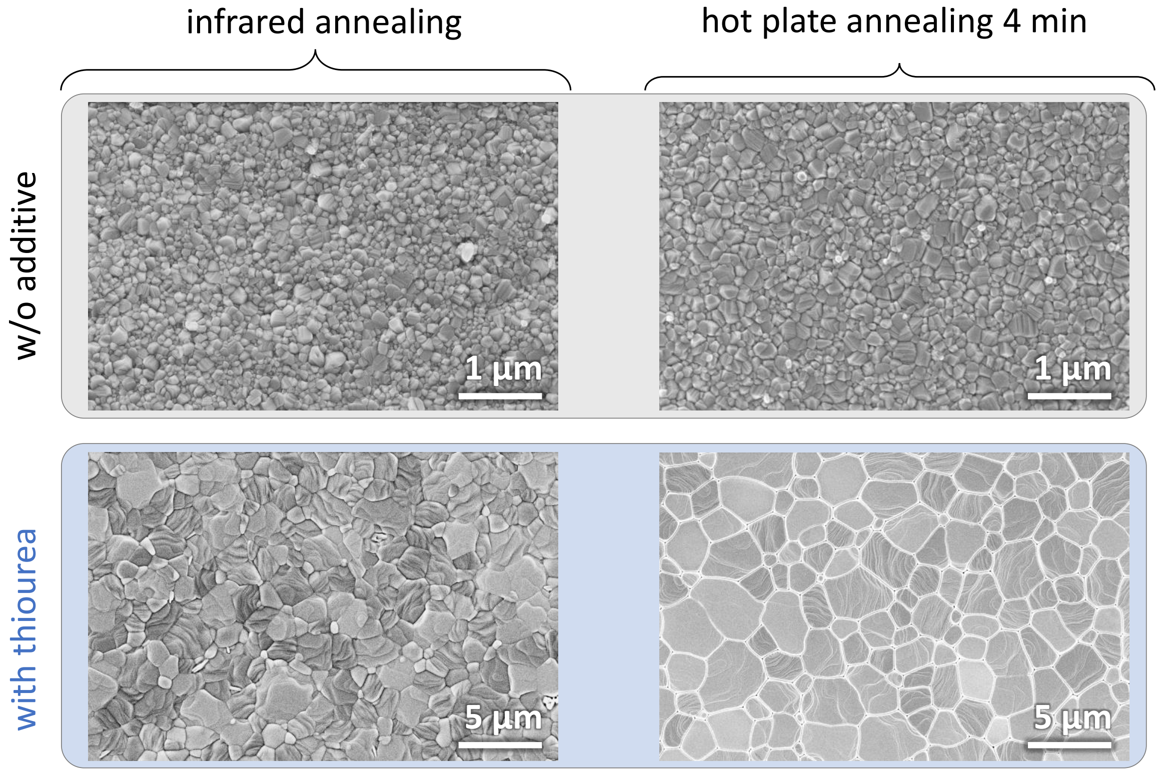

Figure S14: Comparison of $MAPbI_3$ layers employing 0.1 M of thiourea annealed either by infrared lamp or on the hot plate for 4 minutes. The annealing time on the hot plate was adapted to the experimental conditions during the GIWAXS measurement. While again being slightly different in grain morphology, the grain size and the impact of thiourea on the grain size appears comparable in both scenarios.

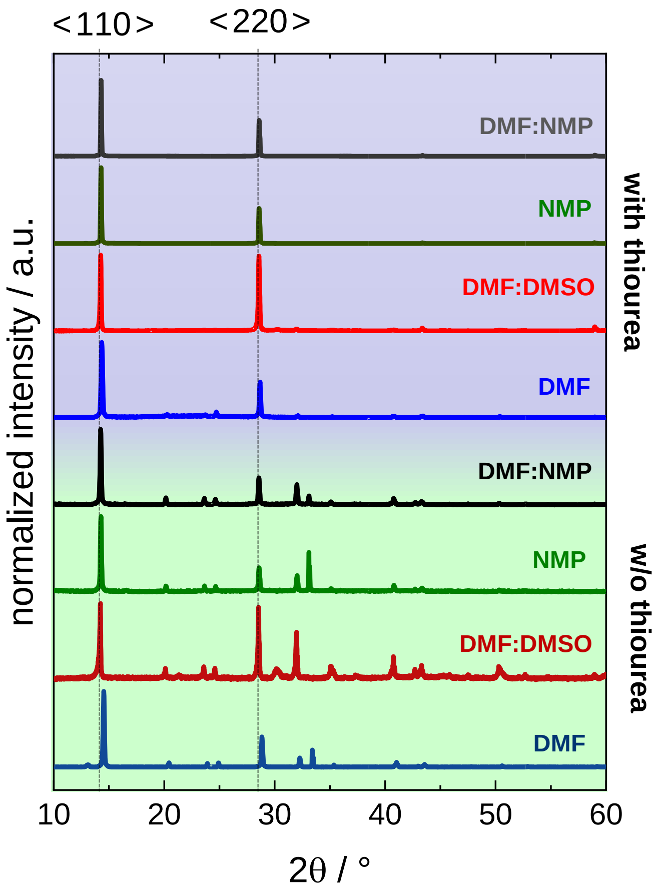

Figure S15: XRD spectra of $MAPbI_3$ thin films deposited from 1 M precursor solutions by gas-quenching (SEM and AFM images can be found in Fig. S8). Note that XRD is probing the direction perpendicular to the substrate in the applied thin film geometry, which is why the well-oriented texture of thin films grown with thiourea additive leads to only peaks of the ⟨110⟩ lattice plane family being visible, while in the corresponding thin films with randomly oriented crystallites, signals from other lattice planes are observed as well.

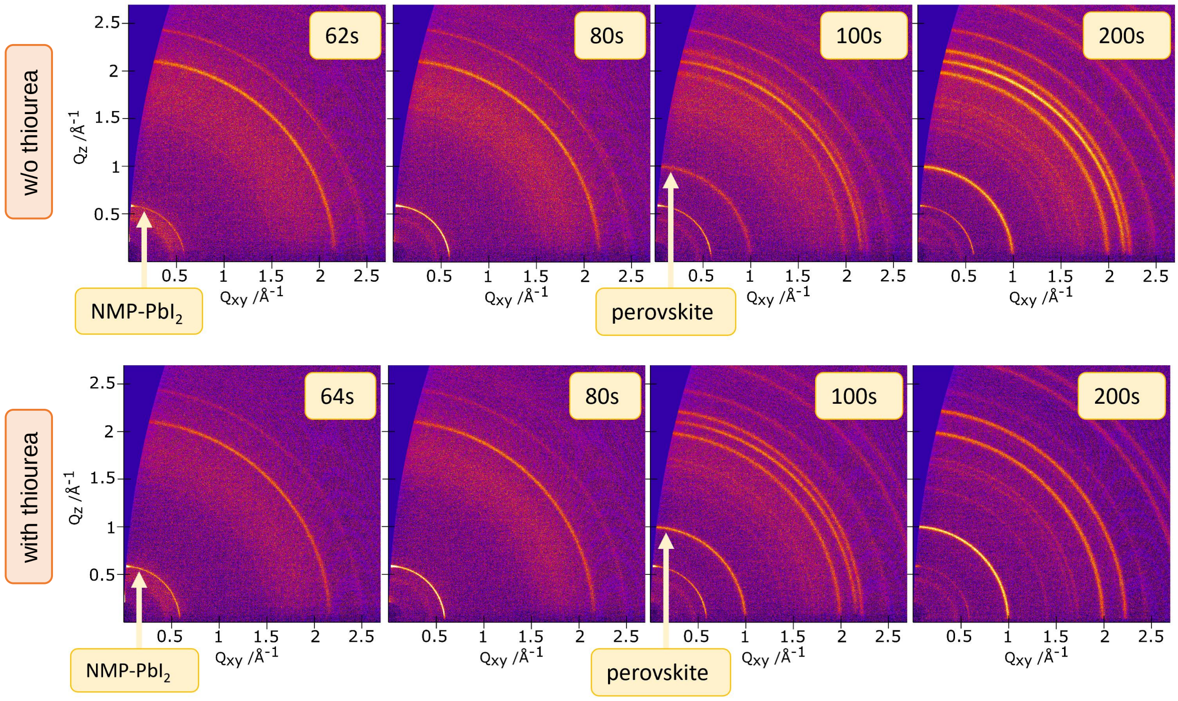

Figure S16: Reciprocal space maps at selected time instances during spin-coating to show the evolution of crystal phases. Top row: $MAPbI_3$ without additive, Bottom row: $MAPbI_3$ with thiourea (source for Fig. 5a, b).

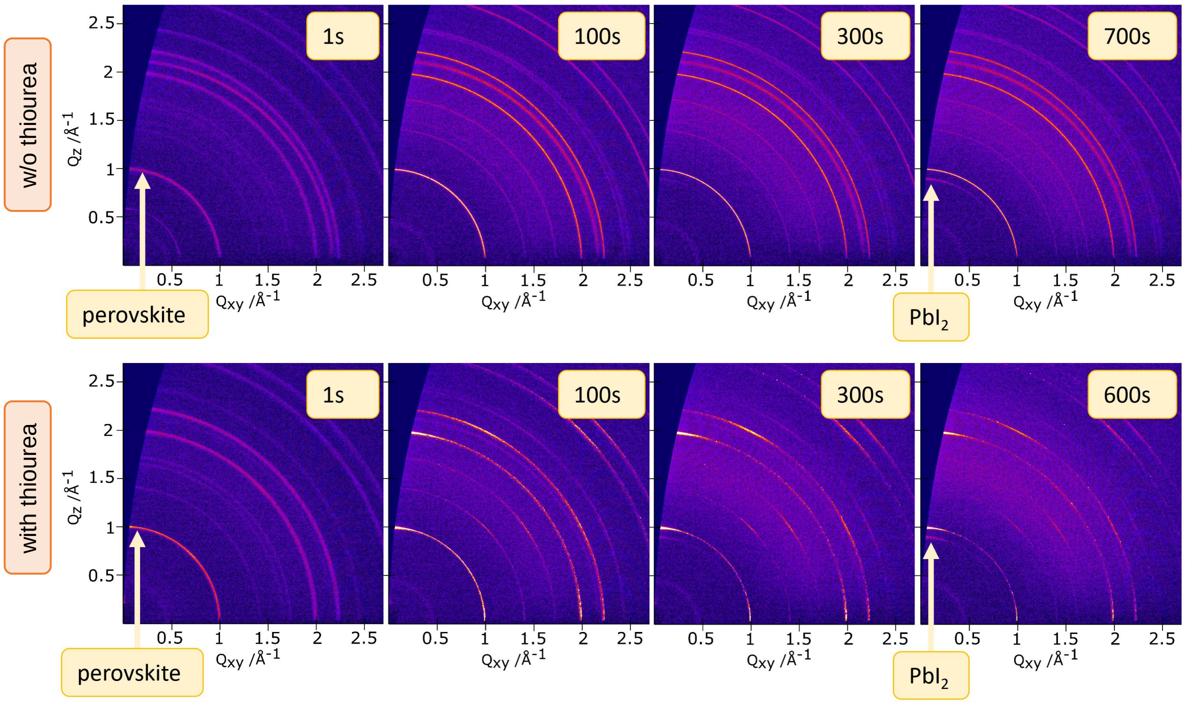

Figure S17: Reciprocal space maps at selected time instances during thermal annealing to show the evolution of crystal phases. Top row: $MAPbI_3$ without additive, Bottom row: $MAPbI_3$ with 0.1 M thiourea additive (source for Fig. 5d, e).

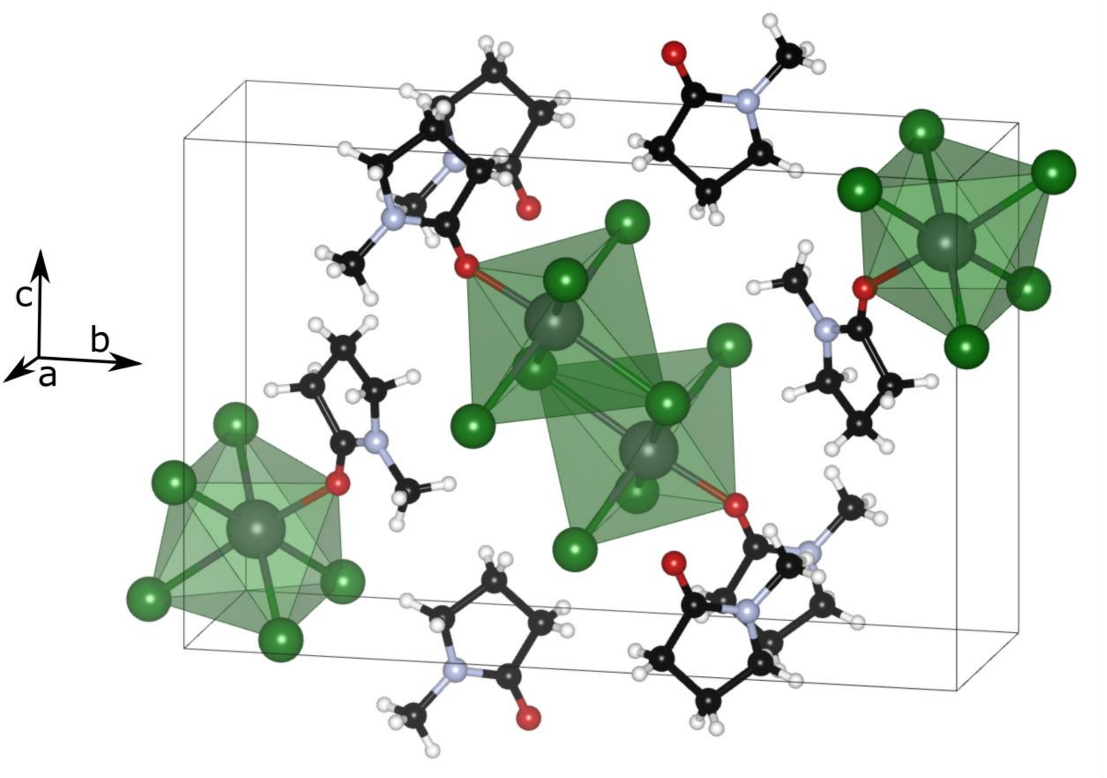

Figure S18: Crystal structure of PbI_2·NMP rendered from ref [2]. Green spheres: iodide, larger grey spheres: lead, small black spheres: carbon, small blue spheres: nitrogen, small red spheres: oxygen, smallest white spheres: hydrogen. Green octahedra indicate the pre-formed PbI_6 motif.

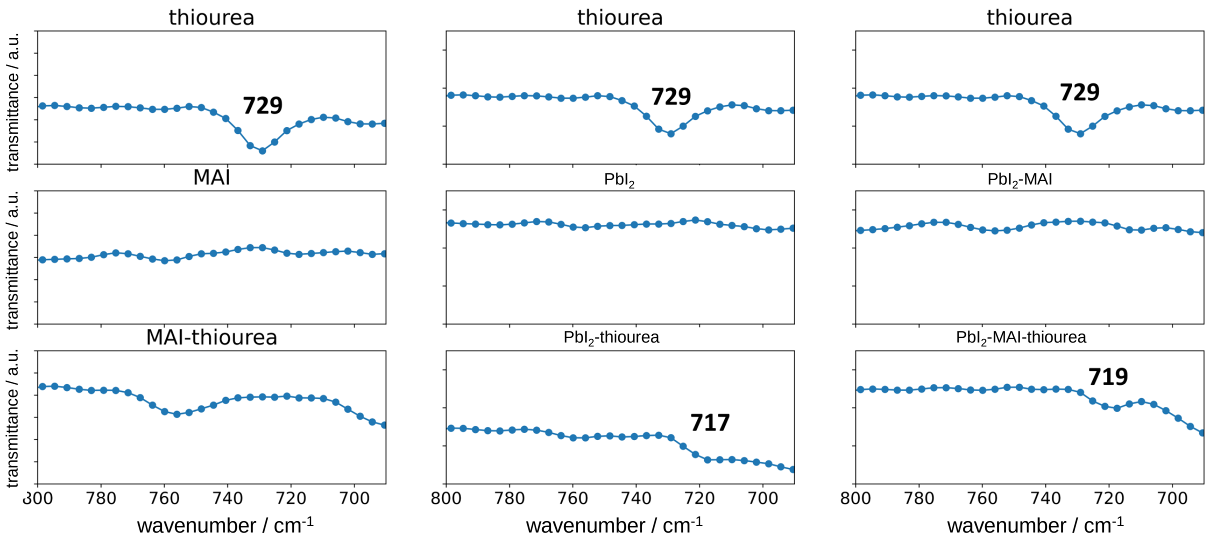

Figure S19: FTIR data (zoom on the C=S stretching of the thiourea molecule) of MAI, PbI_2 and $MAPbI_3$ films deposited from precursor ink with and w/o 0.1 M of thiourea additive.

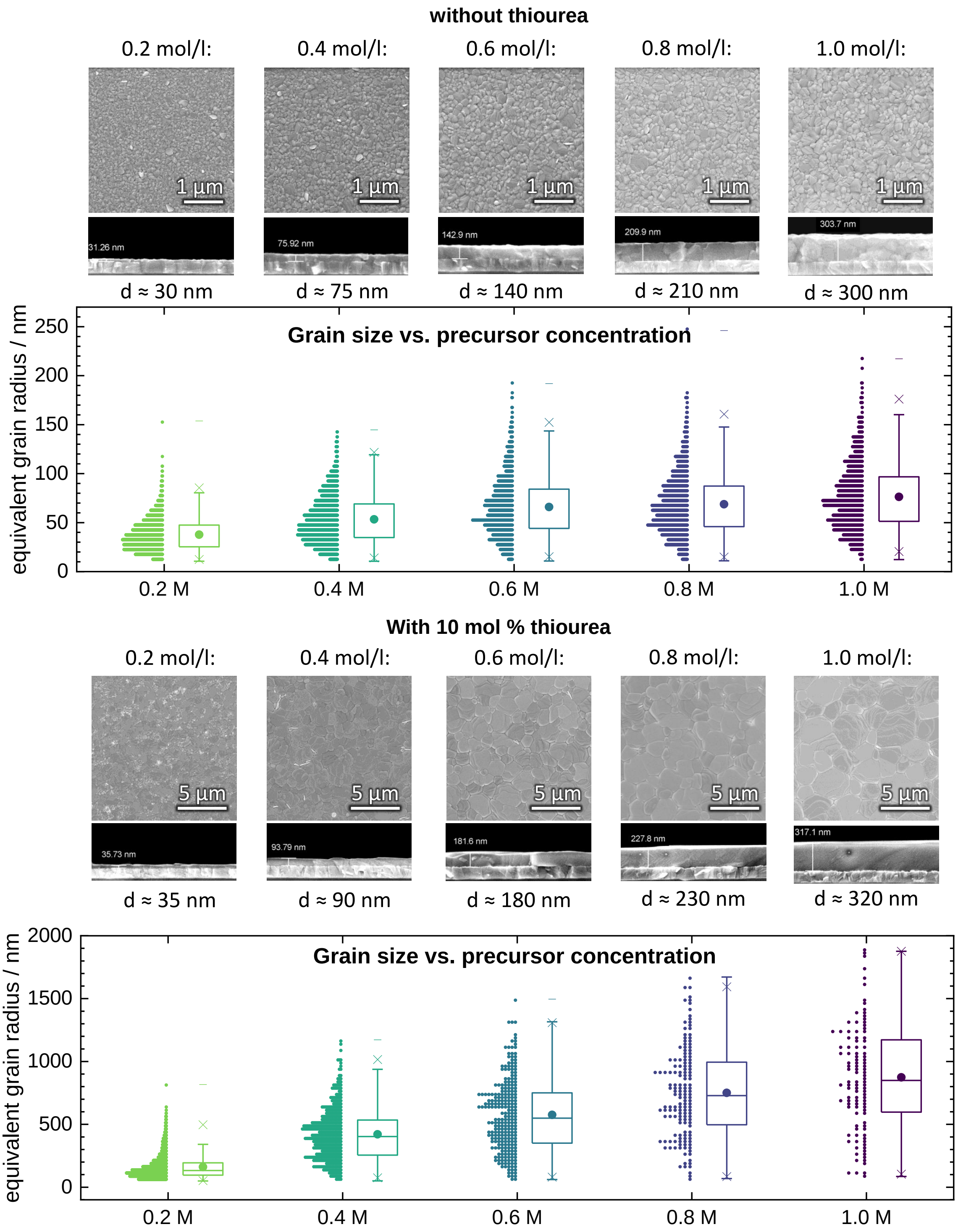

Figure S20: $MAPbI_3$ grain sizes with and without 10 mol-% thiourea at different precursor concentrations illustrating the consistency of the grain-size increase of almost an order of magnitude over a wide range of precursor concentrations. Please note that the increasing precursor concentration increases the film thickness and that the coarsening slows down when the average crystal size approaches the film thickness.[3,4]

Supplementary Note 2

For monitoring the crystallite growth during the spin-coating and annealing procedures by GI-WAXS, an easily trackable parameter was required. Scherrer analysis,[5] as is often used to measure the size of crystalline domains, was not applicable, due to its theoretical and practical limits to domain sizes of about 10 – 100 nm. Thus, peak width is an unreliable indicator for crystallite size and film formation in the thin films containing the thiourea, or any other, additive, where these crystallite sizes may be exceeded. However, this allowed to exploit the finite size of the probing volume creating a "grainy" diffraction pattern (visualized in Fig. S21). Every single crystalline coherently scattering domain hit by an X-ray beam will create one diffraction spot for a given reflection (e.g. the <110> signal). For a considerably sized sample volume with many small crystallites oriented randomly, a large number of single diffraction spots along the circular direction will create a ring-shaped diffraction pattern on the area detector. For a smaller number of domains hit by an X-ray beam, the number of single diffraction spots within the ring-shaped feature will decrease and instead of a smoothly uniform distribution of azimuthal intensity, the pattern will look "grainy". Under the assumption that the illuminated volume, as well as the amount of crystalline material within that volume stays constant, a coarsening of the diffraction pattern, reflecting a reduction of the number of crystalline domains must consequentially imply an increased size of those crystallites.

As a parameter for tracking this coarsening of the diffraction pattern during the thin film deposition, the root-mean-squared azimuthal intensity variation of the strongest perovskite diffraction signal was used, which is a common measure for the roughness of any curve and thus was chosen to represent the "graininess" of the diffraction pattern. In Fig. S21e and f, the azimuthal intensities are displayed as a function of time, where the increase in azimuthal intensity fluctuation is visualized by an increased "stripiness" of the intensity. The chosen diffraction signals for the analysis were the <110> signal for tetragonal $MAPbI_3$ and the <100> signal for FA-Cs based materials, both located around q = 1 Å−1.

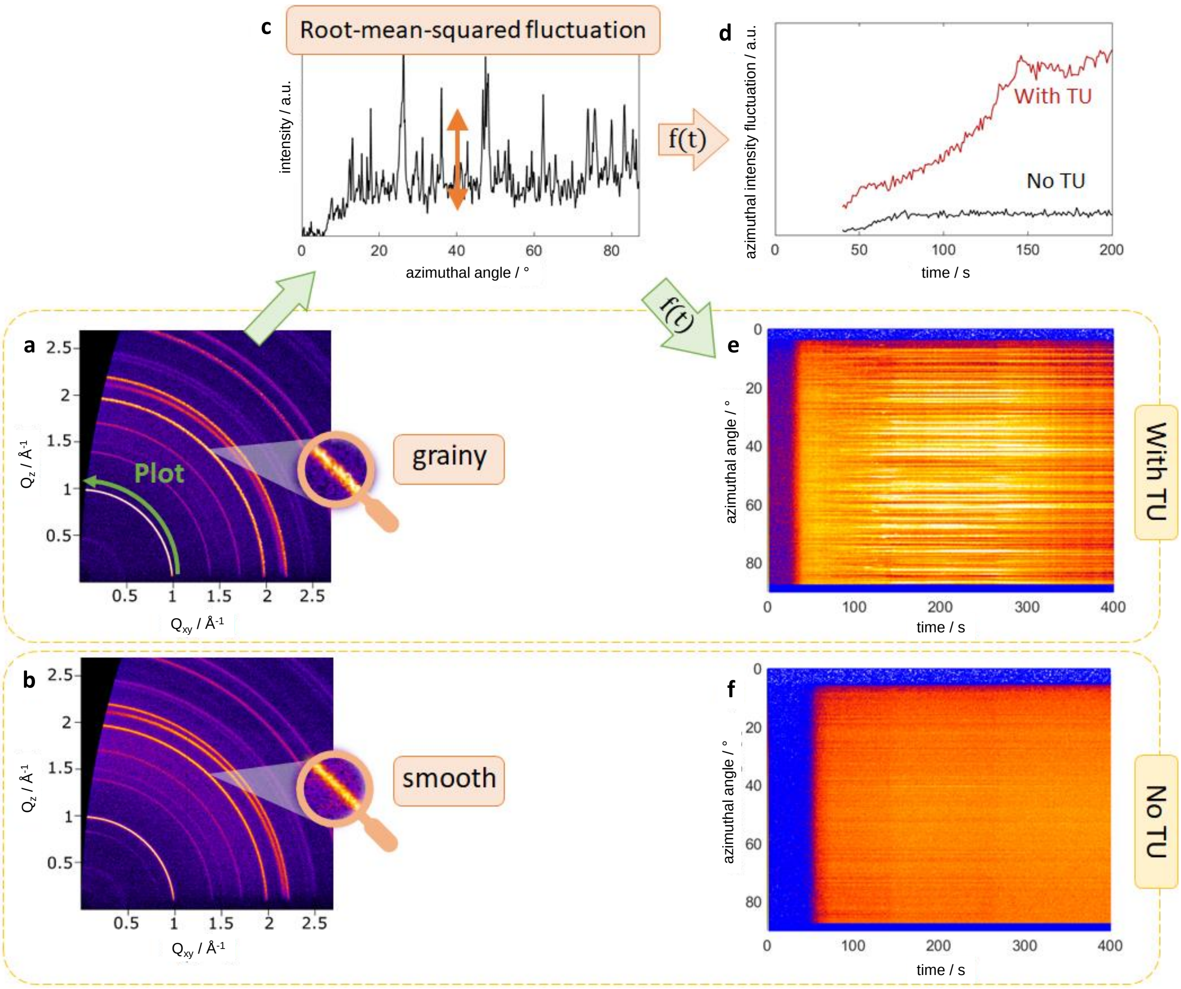

Figure S21: Tracking of crystallite growth via the azimuthal intensity fluctuations of the GIWAXS signal from $FA_{0.94}Cs_{0.06}PbI_3$ perovskite thin films during thermal annealing. **a, b,** Reciprocal space maps for thin films with/without thiourea (TU). **c,** Exemplary azimuthal intensity plot along the perovskite <110> signal. **d,** Root mean squared fluctuation of azimuthal intensity as a function of time during thermal annealing of thin films with/without thiourea. **e, f,** Azimuthal intensities plotted as a function of time.

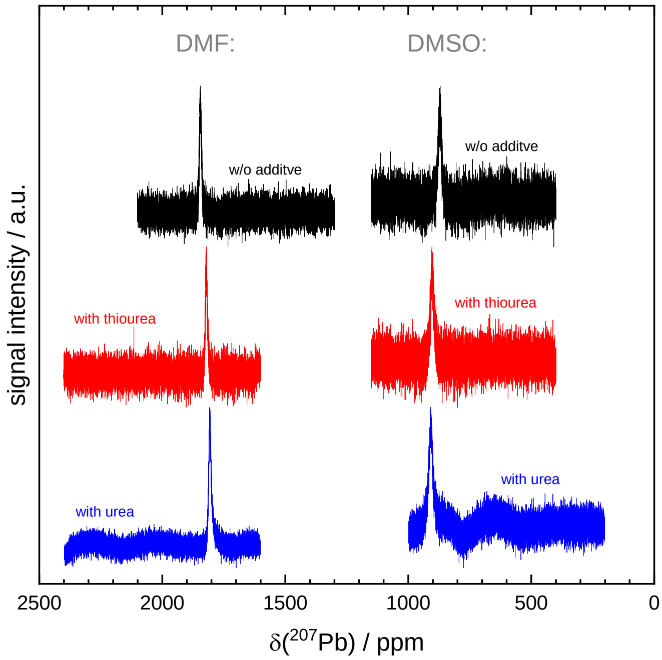

Figure S22: ^{207}Pb-NMR spectra of 1 M perovskite precursor ink solutions in DMF and DMSO, either without additive, with 0.1 M thiourea or with 0.1 M urea additive.

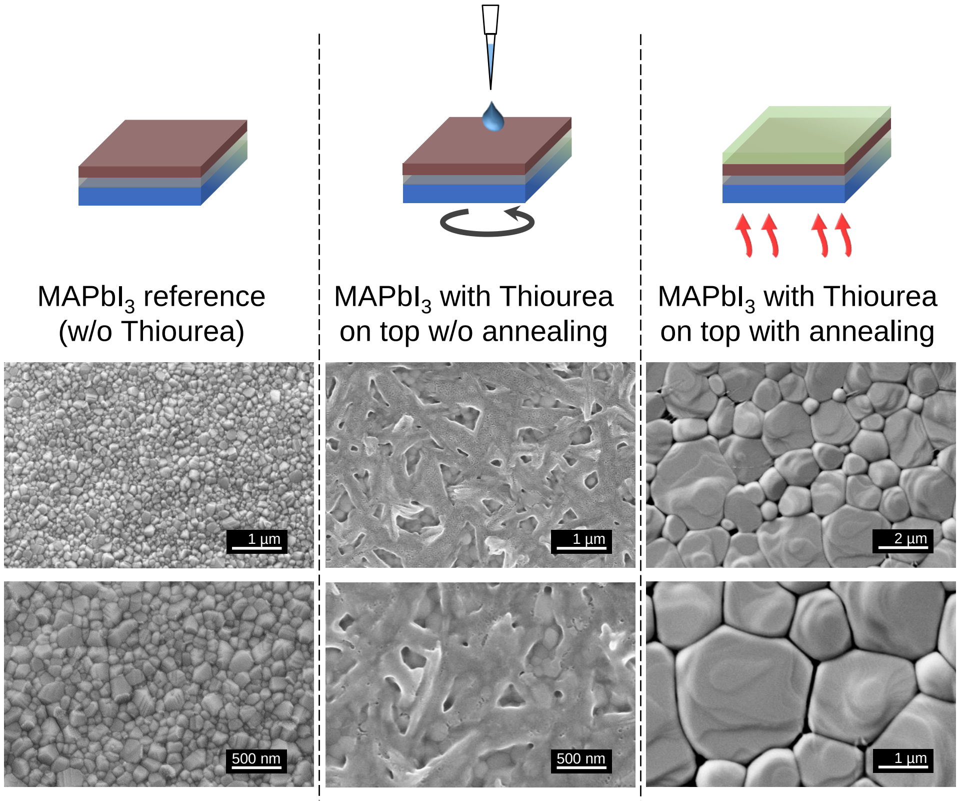

Figure S23: $MAPbI_3$ crystal growth upon deposition of a thiourea solution on top. From left to right: SEM images of pristine, unannealed and annealed samples.

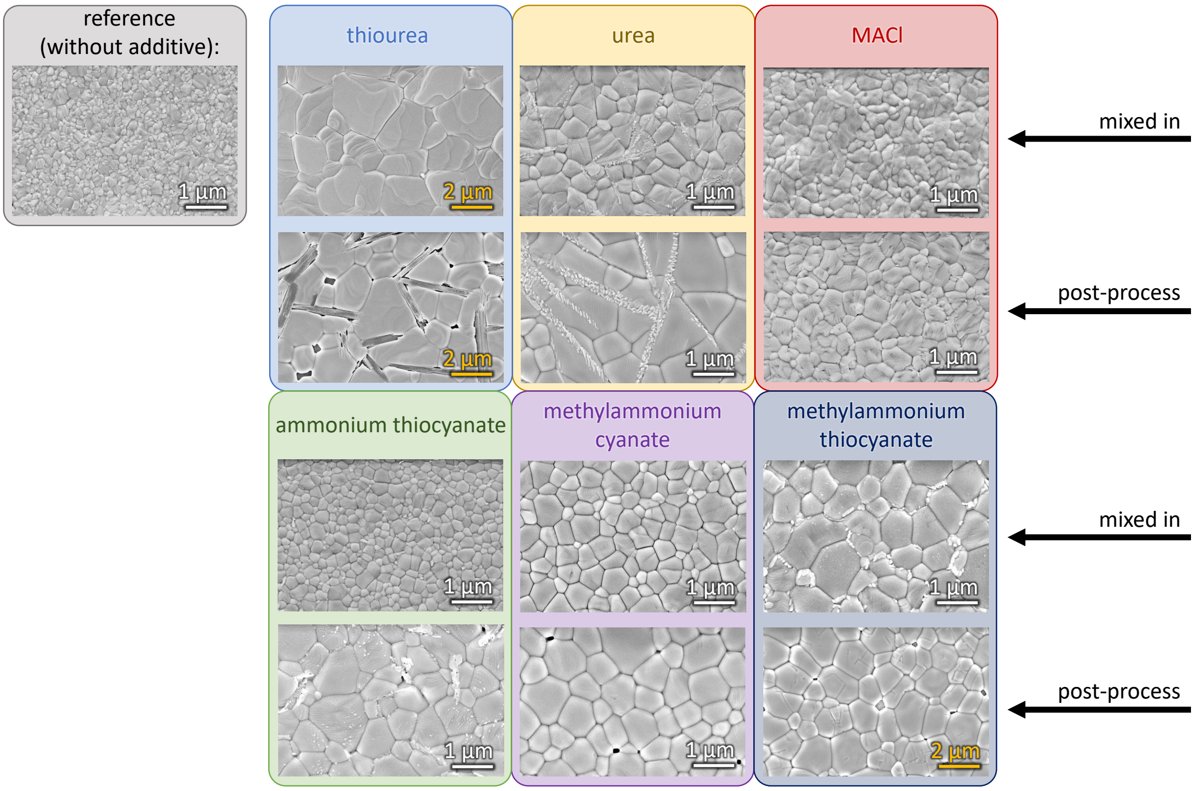

Figure S24: Comparison of popular additives used as a mix-in additive (0.1 M) and as a post-processing crystallization agent for $MAPbI_3$, which clearly shows in the SEM images that all respective crystallization agents increase the grain size regardless of whether they were present during the perovskite nucleation or not.

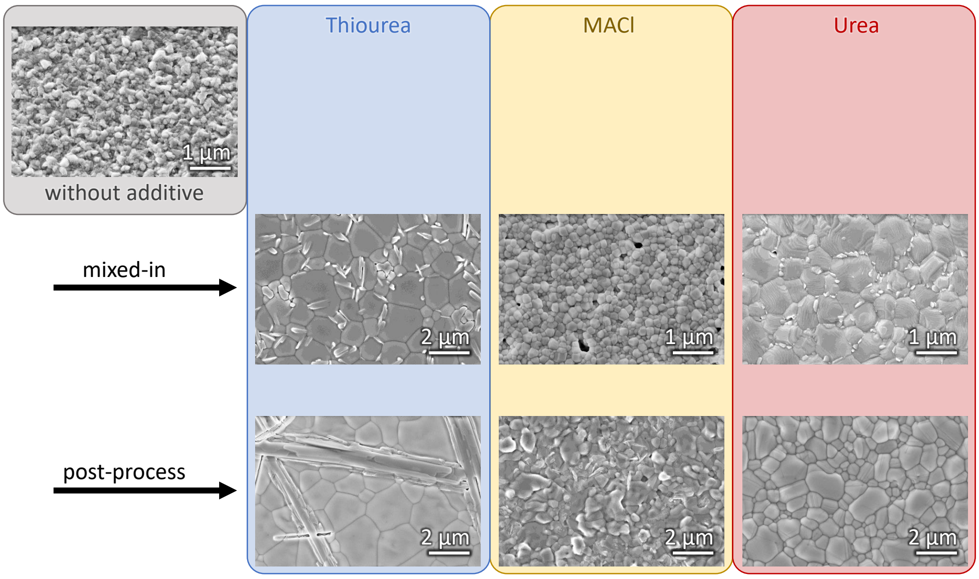

Figure S25: SEM images showing the grain size development with $FA_{0.94}Cs_{0.06}PbI_3$ as the perovskite composition, comparing the additive at 0.1 M mixed into a 1 M precursor solution or used post-process, confirming the validity of our findings also when different perovskite cations are used.

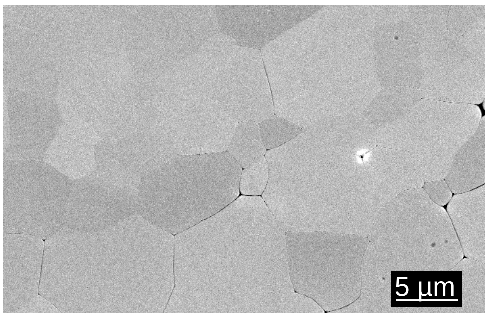

Figure S26: $MAPbI_3$ layer treated with thiourea as shown in Fig. S23, after a follow-up imprinting process at 130 °C. Note the scale bar in comparison to Fig. S23

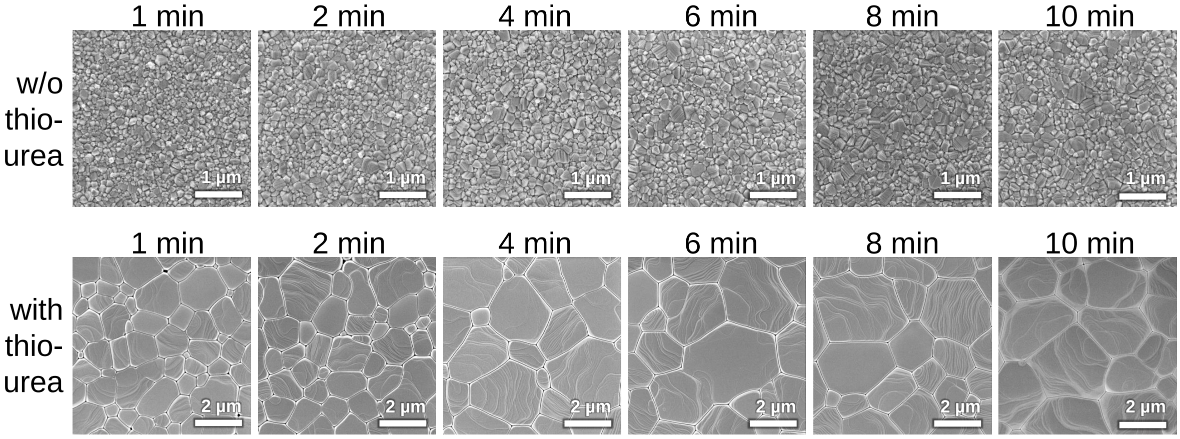

Figure S27: SEM images of $MAPbI_3$ perovskite layers, where the annealing step was interrupted at different stages of the annealing step.

Supplementary Note 3

The crystal size evolution was experimentally observed to be much faster for the samples including thiourea. The hypothesis is that thiourea increases the mobility for the rearrangement of the crystalline interfaces. This is supported by Phase-Field (PF) simulations of grain coarsening with different mobilities. We performed PF simulations for one effective material in 2D and 3D with a computational framework that has been used previously to simulate more complex scenarios.[6] In the current paper, the effect of the mobility on the coarsening rate is investigated. A similar approach was used to investigate the interplay between nucleation and growth.[7] The reader is referred to these previous works for more details.

The state of the system is described by a crystalline order parameter Φ and a marker field θ. The crystalline order parameter can vary from 0 (amorphous state) to 1 (crystalline state). Each crystal is assigned a different marker value in order to distinguish them and handle the grain boundaries. The Gibbs free energy G used to describe the energetic contributions in the system reads:

$$G = \int_V \rho \left(\Phi^2 (3 - 2\Phi) \Delta G_V^{\text{cryst}} + \Phi^2 (\Phi - 1)^2 W \right) + \frac{\epsilon^2}{2} (\nabla \Phi)^2 + \Phi^2 (3 - 2\Phi) \frac{\pi \epsilon_{\text{g}}}{2} \delta_{\text{D}} (\nabla \theta) \, \mathrm{d}V$$

with ρ being the material density, $\Delta G_V^{\text{cryst}}$ defining the energy gain upon crystallization ($\Delta G_V^{\text{cryst}} = L_{\text{fus}}\left(\frac{T}{T_{\text{m}}} - 1\right)$, L_{fus}: enthalpy of fusion, T: temperature, T_{m}: melting temperature), W the height of the energy barrier upon crystallization, ϵ the surface energy between crystalline and amorphous phase and ϵ_{g} the grain boundary energy. The term $\delta_{\text{D}}(\nabla\theta)$ is equal to 1 if a difference in the marker field θ is present and zero otherwise and therefore only active at grain boundaries.
The system evolves according to the stochastic Allen-Cahn equation and leads to the minimization of the energy of the whole system,

$$\frac{\partial \Phi}{\partial t} = -M\frac{\nu_0}{RT}\left(\frac{\partial \Delta G_V}{\partial \Phi} - \nabla\left(\frac{\partial \Delta G_V}{\partial\left(\nabla \Phi\right)}\right)\right) + \zeta$$

with M being the mobility of the solid amorphous interface, ν_0 the molar volume, R the gas constant, and ζ an uncorrelated Gaussian white noise. The noise term is the driving force for coarsening in the system. There is no differential evolution equation for the marker field θ. For simplicity, a grid point gets a marker value assigned/removed if the crystalline order parameter exceeds/falls below the threshold t_Φ. The assigned value is the one of the nearest crystal.[8]

Table S1: Input parameters for the simulations.

Parameter	Value	Unit	Parameter	Value	Unit
ρ	4000	$\text{kg} \cdot \text{m}^3$	ϵ	10^{-4}	$\sqrt{\text{J}} \cdot \text{m}^{-1}$
W	$18.75 \cdot 10^3$	$\text{J} \cdot \text{kg}^{-1}$	ϵ_{g}	0.4	$\text{J} \cdot \text{m}^{-2}$
L_{fus}	$12.5 \cdot 10^3$	$\text{J} \cdot \text{kg}^{-1}$	L_{fus}	$1.5 \cdot 10^{-4}$	$\text{m}^3 \cdot \text{mol}^{-1}$
T	330	K	t_Φ	0.6	1
T_{m}	10	K	M	varies	s^{-1}
grid spacing	10	nm	grid size	10.24×10.24	$\mu\text{m} \times \mu\text{m}$

The experimental data suggest that grain growth proceeds slower than $r \sim t^{1/2}$ or even almost stagnates. There are different possible reasons for this: First, in thin films, the transition from 3D to 2D growth, when the crystals extend from the top to the bottom of the film, and grain grooving at the film surface might be sources of growth stagnation.[8–11] In these cases, however, growth stagnation then occurs when the radii of the crystals are about half of the film height, as shown in Fig. S28. This would be a radius of approximately 150 nm in our case and since we obtained micrometer-sized grains, these processes are certainly not the limiting ones in the present work. Second, growth stagnation could also be related to the removal of the mobility-promoting additive thiourea with time, but it has been checked that thiourea remains in the system even after the longest heating times as shown in Fig. S19.

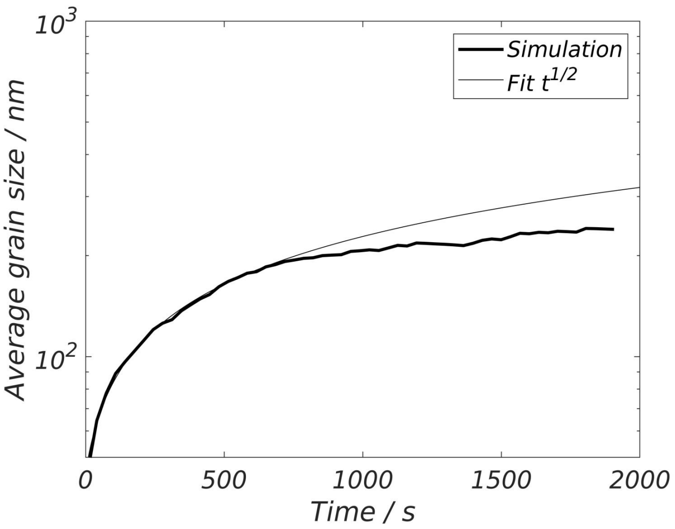

Figure S28: 3D Phase-field simulation of grain coarsening in a 300 nm thick film (normal grain growth). The initial average grain size at time t_0 is $r_0 = 50$ nm. Until about 600 s and an average radius of 180 nm, the simulation data can be fitted using the standard law for normal growth $r = \left[{r_0}^2 + k(t - t_0)\right]^{1/2}$. At this point all grains form columnar structures extending from the substrate to the film surface and the coarsening rate drops significantly.

Third, perovskite films might follow the very common “abnormal” grain growth behavior, which is encountered in many material systems.[12] This “abnormal grain growth” leads to the mean grain size following an asymptotic behavior $r \sim t^n$ with $n < 0.5$ and even n decreasing with time. This is generally thought to be related to the fact that the mobility (and/or interfacial energy) is significantly lower for some of the grain boundaries (see Fig. S29), and it has been shown that even a small amount of slow grain boundaries could lead to full stagnation of growth.[13] Such an abnormal growth process leads to a more widespread crystal size distribution with some grains sometimes growing catastrophically while others remain very small (see Fig. S30). As we would expect the presence of thiourea and also the nature of the grain boundaries themselves to be subject to some spread, we used a portion of 10 % low-mobility grain boundaries.

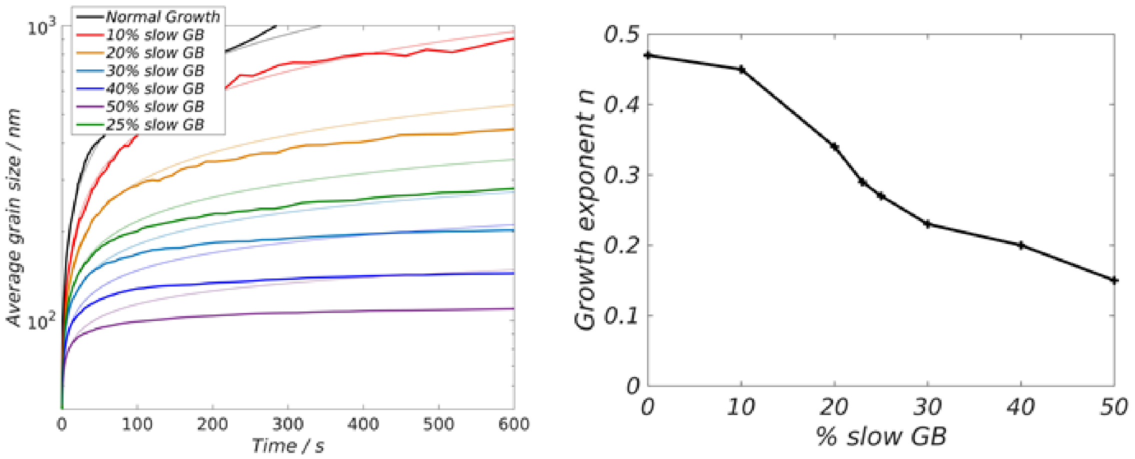

Figure S29: Simulation of abnormal growth with 10 % to 50 % grain boundaries with negligible mobilities. (Left) Average grain size (bold curves) from simulation data and (thin curves) fits to the data using $r = \left[r_0{}^{1/n} + k(t - t_0)\right]^{1/n}$ over more than 2 decades of time for $t < 30$ s. The discrepancy between fit and simulation at late times, for large amount of slow grain boundaries, indicates that the growth exponent decreases with time. (Right) Growth exponent obtained from the fits in the initial coarsening phase ($t < 30$ s).

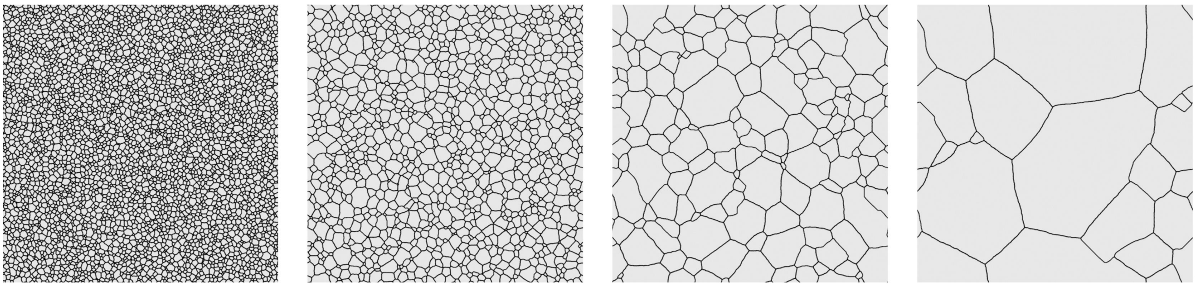

Figure S30: Snapshots of the simulated film morphologies (Top) After 1 s, 10 s, 100 s and 1000 s of grain growth, for 10 % slow grain boundaries. The box size is 10 µm × 10 µm.

Supplementary Note 4

$MAPbI_3$ and $MAPbI_3$ + 5 % thiourea samples were thoroughly characterized using SEM, PXRD, and NQR to evaluate their structural and morphological properties.

The pseudo-radii of individual powder grains were determined by measuring the grain areas from SEM images (Fig. S31a,b). The average particle pseudo-radii for $MAPbI_3$ and $MAPbI_3$ + 5 % thiourea were approximately 1.3 µm and 1.1 µm, respectively. This roughly resembles the saturation grain size we obtained for our high mobility coarsening in Fig. 5c. We speculate that the milling process with milling agent provides sufficient mobility to reach this state.

PXRD analysis revealed that both $MAPbI_3$ and $MAPbI_3$ + 5 % thiourea exhibited similar tetragonal crystal structures (Fig. S31c). However, the addition of thiourea resulted in narrower diffraction peaks, indicating an increase in crystallinity.

NQR spectroscopy of quadrupolar halides is highly sensitive to the local environment surrounding the halide nuclei, making it an effective tool for probing defect density within the perovskite

lattice.[14] Both the absolute signal intensity and, in particular, the half-width of the NQR signals serve as qualitative indicators of defect densities.[15,16] The ^{127}I NQR spectra of $MAPbI_3$ with and without thiourea are shown in Fig. S32, with corresponding full-width at half-maximum (FWHM) values of 10.31 MHz and 15.05 MHz, respectively. These results suggest a slightly lower defect density in the sample containing thiourea.

To monitor the evolution of halide ion exchange between powder grains of $MAPbI_3$ + $MAPbBr_3$, $MAPbI_3$ + $MAPbBr_3$ + 5 % thiourea, and $MAPbI_3$ + $MAPbBr_3$ + 10 % thiourea, a series of 1D ^{207}Pb-NMR spectra were recorded at 80 °C as shown in Fig. 5d. The experimental procedure and data processing followed the method described in ref [17].

As shown in Fig. 5d (top), in the beginning, the ^{207}Pb MAS NMR spectrum showed two main signals, which correspond to [PbI_6] and [$PbBr_6$] environments in the two perovskite phases $MAPbI_3$ and $MAPbBr_3$. As we annealed the powders at 80 °C, additional signals started to appear and grew between the original two, which are corresponding to [$PbI_{6-x}Br_x$] environments forming within mixed halide perovskite $MAPbI_{3-x}Br_x$ phases. This formation of a solid solution resulted from the diffusion of Iodide (I^-) and Bromide (Br^-) ions between the $MAPbI_3$ and $MAPbBr_3$ powders. During the annealing process, the signals from $MAPbI_3$ and $MAPbBr_3$ continuously decreased, while the mixed perovskite phase increased. The spectra were fitted with seven Gaussian functions, each with a fixed FWHM and position corresponding to [PbI_6], [PbI_5Br_1], [PbI_4Br_2], [PbI_3Br_3], [PbI_2Br_4], [PbI_1Br_5] and [PbI_6] following our previously published approach.[13] To quantify the formation of mixed halide phases the integrals of [PbI_5Br_1], [PbI_4Br_2], [PbI_3Br_3], [PbI_2Br_4], [PbI_1Br_5] were summed for each time step. In Fig. 5d (bottom), the evolution of integrals of the mixed halide environments [$PbI_{6-x}Br_x$] (where $0 < x < 6$) as a function of annealing time at 80 °C is shown for $MAPbI_3$ + $MAPbBr_3$, $MAPbI_3$ + $MAPbBr_3$ + 5 % thiourea, and $MAPbI_3$ + $MAPbBr_3$ + 10 % thiourea. The resulting data was fitted using the Johnson-Mehl-Avrami-Kolmogorov model,[18] with the fitting parameters provided in Table S2.

In this model, k represents the reaction rate, while n describes the diffusion process in an empirical way.[18] As we have no detailed information on the diffusion process itself, n was fixed between 0.2 – 0.25, fitting the experimental data best for all sample sets. As the value n is similar between the three sample sets, the reaction rate k for the formation kinetics of the mixed halide perovskite phase, can directly be compared.

Analogously, the evolution of halide ion exchange between powder grains of $MAPbI_3$ + $MAPbBr_3$, $MAPbI_3$ + $MAPbBr_3$ + 10 % MACl (batch 2) was monitored via ^{207}Pb MAS NMR spectroscopy at 80 °C and evaluated (Fig. S33). The resulting kinetic curves were fitted using the Johnson-Mehl-Avrami-Kolmogorov model,[18] with the fitting parameters provided in Table S3. It is important to note that ion diffusion, and consequently the mixing rate, is influenced by both the physical and chemical properties of the powders, such as particle size and defect density, as well as the characteristics of the physical powder mixtures. Since these properties can vary between different synthesis batches, the extracted k values should always be compared relatively to the reference mixture from the same batch.

The quantitative ^{1}H MAS NMR spectra of $MAPbI_3$ with 5 % thiourea recorded with 62.5 kHz and

20 kHz spinning rate both display two prominent signals at 3.3 ppm and 6.3 ppm corresponding to the methylammonium cation ($-CH_3^+$) and ($-NH_3^+$), respectively. Additionally, a broad signal is resolved at ∼7 ppm in the high-resolution ^{1}H MAS NMR spectrum (ν_{rot} = 62.5 kHz, Fig. S35a) corresponding to the amine group of thiourea, which due to its broad nature and low intensity seems invisible in the spectrum recorded with lower spinning speed (Fig. S35b). In order to enhance the relative intensity of the thiourea proton signal compared to the perovskite signals the recycle delay was reduced (d_1 = 1 s, Fig. S35c).

To gain further insight into the behavior of thiourea at elevated temperatures, and ultimately the mixing mechanism, variable temperature (VT) ^{1}H MAS NMR experiments were conducted at temperatures between 30 °C and 80 °C. Fig. S35d – h displays a zoom of the VT ^{1}H MAS NMR spectra with reduced recycle delay (1 s) at temperatures between 30 and 80 °C. The increase in temperature results in a significant narrowing of the thiourea signal indicating a partial averaging of dipolar interactions due to an increase in molecular motion of the thiourea molecules. Thus, at elevated temperatures the thiourea molecules may detach from perovskite grain interfaces, leaving unsaturated defect sites behind. The evolution of the full width at half maximum (FWHM) of the thiourea signal as a function of temperature is displayed in Fig. S35i.

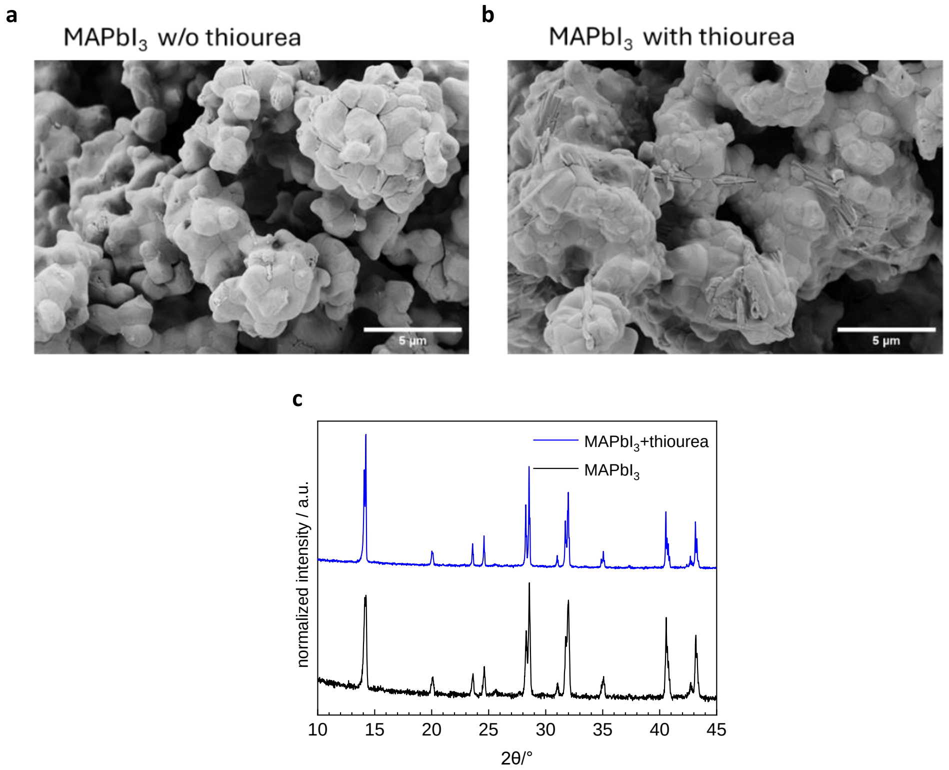

Figure S31: a, SEM images of $MAPbI_3$, and **b,** $MAPbI_3$ with 5 mol-% thiourea. **c,** PXRD patterns comparing $MAPbI_3$ and $MAPbI_3$ with 5 mol-% thiourea.

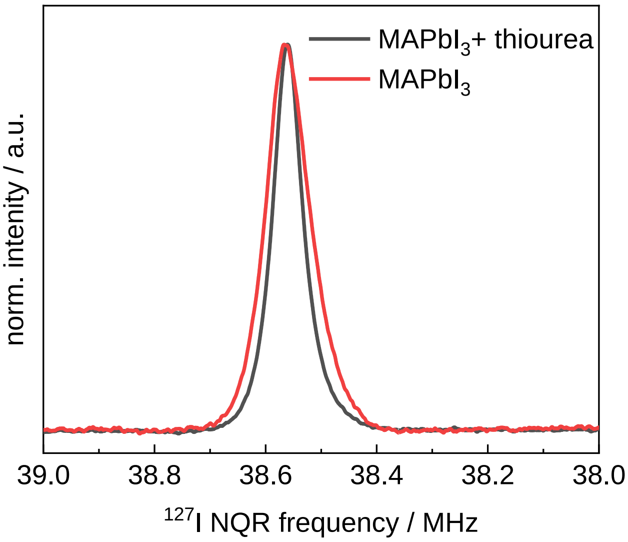

Figure S32: ^{127}I NQR spectra of the $MAPbI_3$ and $MAPbI_3$ + thiourea.

Table S2: Fitting parameters for the build-up curve corresponding to the phase formation of $MAPbI_{1.5}Br_{1.5}$ extracted from the formation of $[PbI_{6-x}Br_x]$ environments, as shown in Fig. 5d.

starting sample composition	A	k / min^{-1}	n
$MAPbBr_3$ + $MAPbI_3$	1	$2.7 \cdot 10^{-3}$	0.25
$MAPbBr_3$ + $MAPbI_3$ + 5 mol-% thiourea	1	$2.6 \cdot 10^{-2}$	0.20
$MAPbBr_3$ + $MAPbI_3$ + 10 mol-%	1	$1.2 \cdot 10^{-1}$	0.20

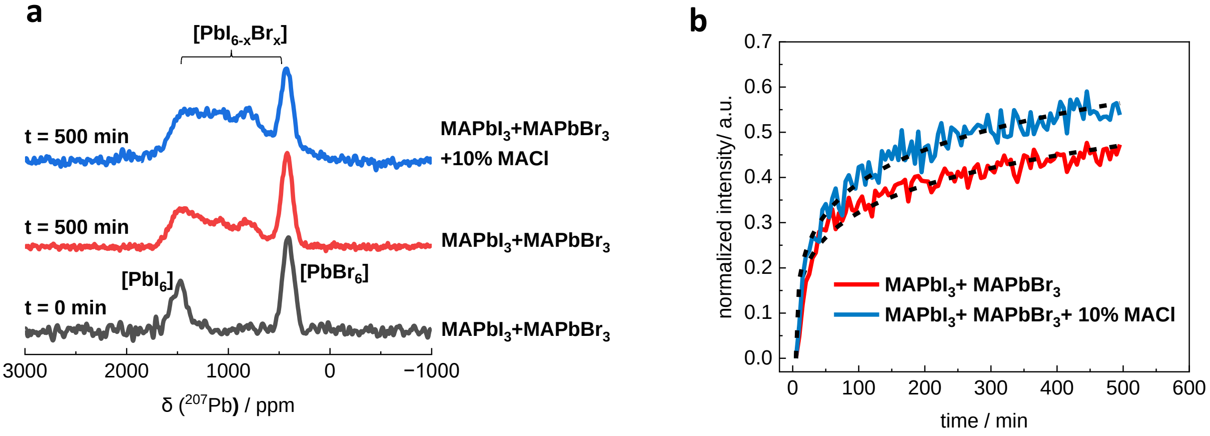

Figure S33: a, ^{207}Pb MAS NMR spectra of a physical mixture of $MAPbI_3$ + $MAPbBr_3$ and $MAPbI_3$ + $MAPbBr_3$ + 10 % MACl before and after annealing at 80 °C for 500 min. **b,** Corresponding normalized intensity of the formation of $[PbI_{6-x}Br_x]$ environments in mixed halide perovskite phases as a function of annealing time at 80 °C. Fits are based on the Johnson-Mehl-Avrami-Kolmogorov model and fit parameters are summarized in Table S3.

Table S3: Fitting parameters for the build-up curve corresponding to the phase formation of $MAPbI_{1.5}Br_{1.5}$ and $MAPbI_{1.5}Br_{1.5}$ + 10 mol-% MACl extracted from the formation of $[PbI_{6-x}Br_x]$ environments, as shown in Fig. S33.

starting sample composition	A	k / min^{-1}	n
$MAPbBr_3$ + $MAPbI_3$	1	$4.7 \cdot 10^{-4}$	0.32
$MAPbBr_3$ + $MAPbI_3$ + 10 mol-% MACl	1	$1.2 \cdot 10^{-3}$	0.30

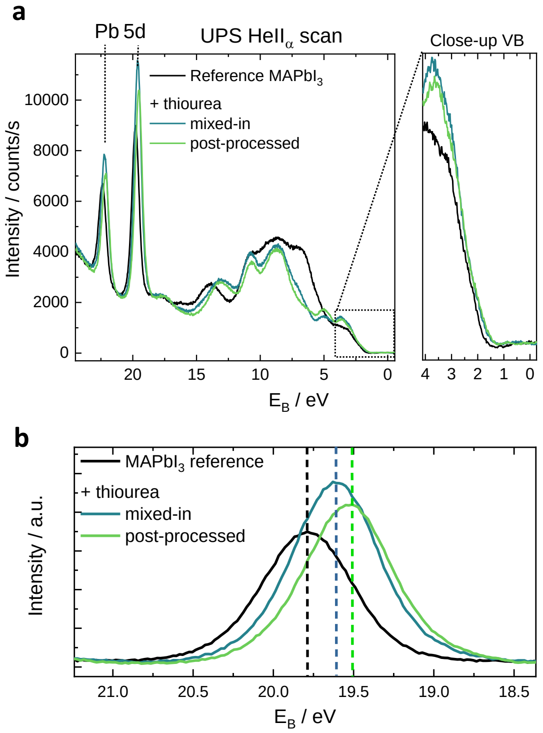

Figure S34: UPS data of the reference $MAPbI_3$ sample, as well as the samples treated with either thiourea or MACl, taken using a monochromatic He IIα excitation. **a,** Shows a wide scan of the VB regions of the reference and thiourea treated samples; the position of the semi-core levels of the Pb5d doublets are indicated. **b,** focuses on the Pb $5d_{5/2}$ peak upon addition and post processing of thiourea. For both the mixed-in and post-treated annealed samples, a clear shift to higher binding energy is evident, indicating an electron donation to the lead orbitals by the additive.

Note that the UPS data of the different measurements was aligned such that the VB onset of all measurements are the same. This was done to eliminate effects of changes in Fermi level position which were present between the different samples. Such a shift in Fermi level also changes the position of the Pb semi-core levels, which would however not be representative for charge transfer between the additive and the Pb atoms. In contrast, the shifts presented here are of the Pb semi-core level with respect to the perovskite VB maximum, and are therefore a clear indication of electron transfer to the surface Pb atoms.

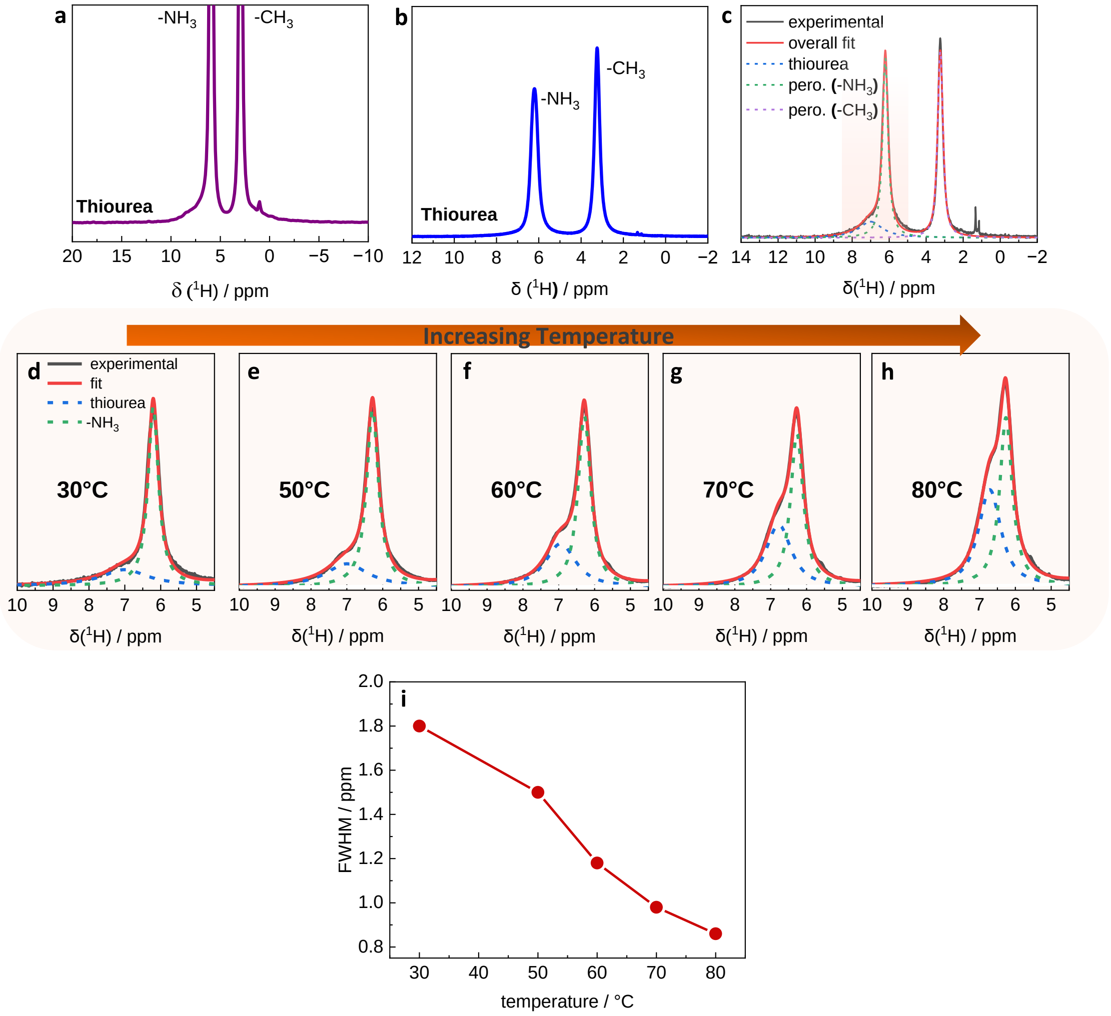

Figure S35: Quantitative ^{1}H MAS NMR spectra of $MAPbI_3$ + thiourea at **a,** 62.5 kHz spinning rate, **b,** at 20 kHz spinning rate. **c,** Non-quantitative ^{1}H MAS NMR spectra of $MAPbI_3$ + thiourea with reduced recycle delay (1 s) at 20 kHz MAS enhancing the thiourea signal at 7 ppm compared to the perovskite signals. **d, – h,** Variable temperature (VT) ^{1}H MAS NMR spectra (recycle delay: 1 s, 20 kHz MAS) focusing on the signals of the perovskite NH_3^+ group and thiourea are shown at **d**, 30 °C, **e,** 50 °C, **f,** 60 °C, **g,** 70 °C, and **h,** 80 °C. The VT ^{1}H MAS NMR spectra highlight the temperature-dependent narrowing of the thiourea signal indicating an increase in thiourea mobility at elevated temperatures. The full width half maximum (FWHM) of the thiourea signal is plotted in **i.**

Supplementary Note 5

Bonding energy

To investigate the interaction strength of molecular additives with MA^+ and I^-, the bonding energy is calculated by placing the molecule, with or without the accompanying ion, in a $15 \times 15 \times 15$ Å^3 vacuum box. The bonding energies between the solvent molecules (DMSO and DMF) and MA^+/I^- are also provided as a reference. The results and corresponding structures are

presented in Table S4 and Fig. S36.

Table S4: Bonding energy of the solvent and additive molecules with either MA^{+} or I^{-}.

molecule	with MA^{+}	with I^{-}
DMF	-1.69	-0.19
DMSO	-1.70	-0.15
urea	-1.61	-0.80
thiourea	-1.45	-0.94

The bonding energies listed in Table S4 suggest that the solvents (DMF, DMSO) as well as the additives (urea, thiourea) tend to have strong bonding interaction with MA^{+}. In contrast to the solvent molecules, both urea and thiourea show a clear preference for I^{-}. Compared to the solvents, these additives exhibit approximately 0.7 eV lower bonding energy, indicating that, unlike the solvents, they can form bonds with I^{-}. This bonding is likely responsible to facilitate the migration of iodide. This supports the hypothesis that thiourea and urea act as ion carriers on the $MAPbI_3$ surface. Furthermore, the bonding energy of thiourea is lower than that of urea by 0.14 eV, which explains why thiourea performs better as an additive than urea in transporting I^{-} ions during the annealing of the $MAPbI_3$ films.

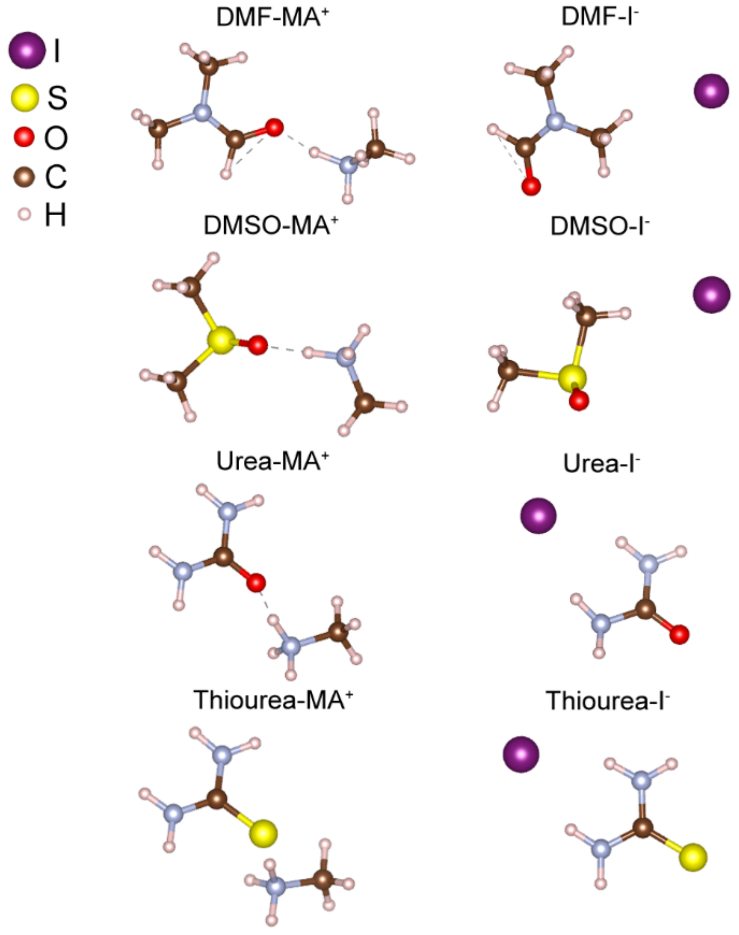

Figure S36: Different pairs of DMF, DMSO, urea or thiourea with MA^{+} or I^{-}.

Electrostatic potential

The electrostatic potential map (Fig. S37) of each molecule-I^- and molecule-MA^+ pair was calculated to gain further insight into the bonding interactions. The molecule alone was also computed to investigate its polarity. The results for DMF and DMSO are also included as references. From the electrostatic potential distribution, it is evident that (thio)urea exhibits stronger polarity compared to DMF and DMSO. In both thiourea and urea, the negative polarity is primarily concentrated around the sulfur and oxygen atoms. As a result, regions further from these atoms exhibit electropositive characteristics. This is characterized by two NH_2 moieties with electropositive characteristic, both interacting with I^-, explaining the strong interaction energies between the two species. In contrast, both solvents exhibit low polarity, showing slight positive polarization of the CH_3 group, and with only one C-H bond interacting with I^-. Therefore, their interaction energy is minimal. Consistent with the bonding energy results, the interaction between (thio)urea and MA^+ is equally strong as those between DMF/DMSO and MA^+, due to the similar interaction strength of negative O/S atoms with the positive NH_3^+.

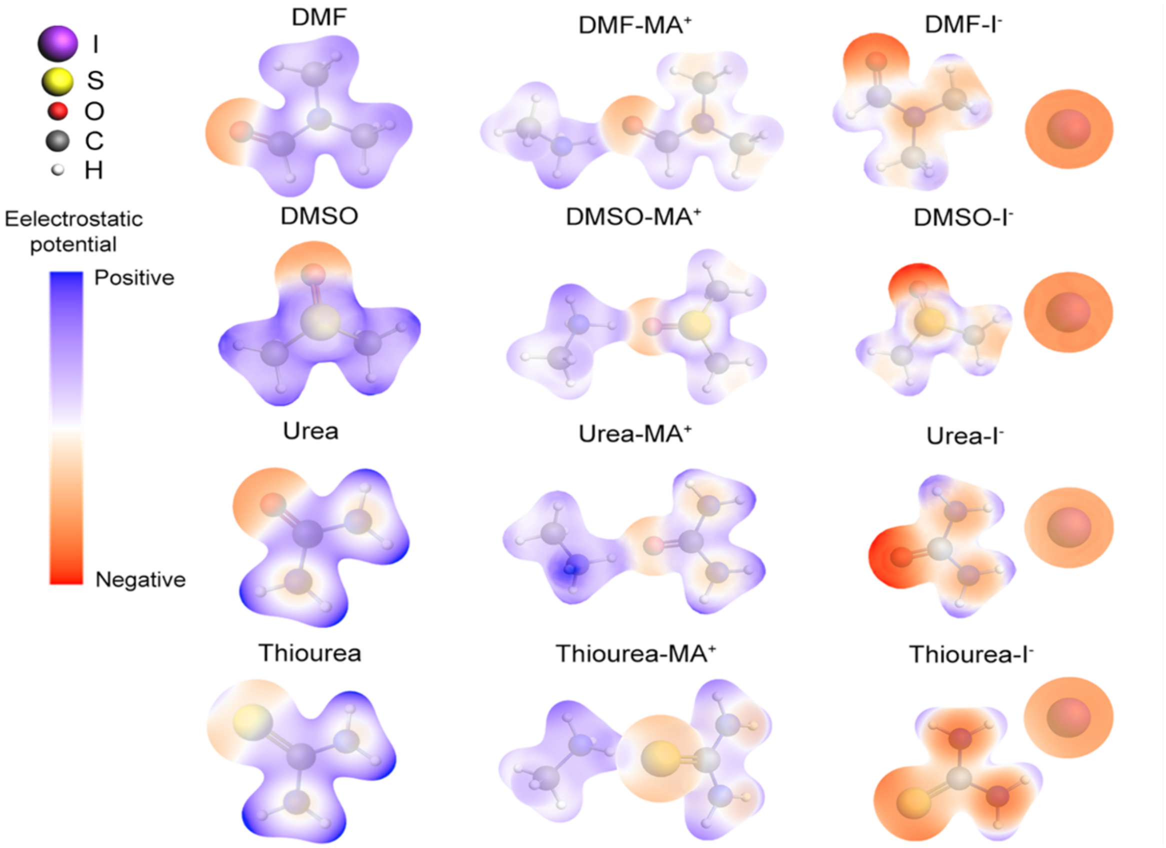

Figure S37: Electrostatic potential distribution of DMF, DMSO, urea, thiourea and their pairs with MA^+ or I^-, color mapping with the potential value, where blue represents the positive potential and red for the negative.

Adsorption Energy

To assess the interaction of urea and thiourea with the $MAPbI_3$ surface, we calculated their adsorption energies on a tetragonal-phase 2×2×1 $MAPbI_3$ slab, while only the additional molecule is allowed to relax during the structural relaxation. The results are presented in Table S5, and the relevant structures are shown in Fig. S38. The adsorption energies of DMF and DMSO are also provided as references.

DMSO exhibits the lowest adsorption energy, followed by urea. The solvent NMP also has a high adsorption energy as −0.73 eV, slightly smaller than urea and DMSO. The adsorption energy of thiourea is much lower than that of urea and DMSO with a value of −0.17 eV. DMF shows a very small adsorption energy, suggesting that it has negligible adsorption on the $MAPbI_3$ surface. The high adsorption energy of urea indicates that it can remain firmly on the surface, which is beneficial for passivating defects. However, when compared to thiourea, the relatively high adsorption energy also means that urea is less likely to desorb from the surface, limiting its mobility. This reduced mobility further restricts the transfer of I^- ions, ultimately diminishing the effectiveness of urea in promoting the formation of larger grains during the annealing process.

Table S5: Adsorption energy of solvent and additives on the $MAPbI_3$ surface.

molecule	adsorption energy / eV
DMF	−0.08
DMSO	−0.96
urea	−0.88
thiourea	−0.17
NMP	−0.73

The structures shown in Fig. 3 suggest that during the adsorption process, the sulfur or oxygen atoms in thiourea and urea can form a bond with the Pb^{2+} cation on the $MAPbI_3$ surface, which may explain the electron donation observed in experiments. This interaction between oxygen or sulfur and Pb^{2+} is also present in the solvent.

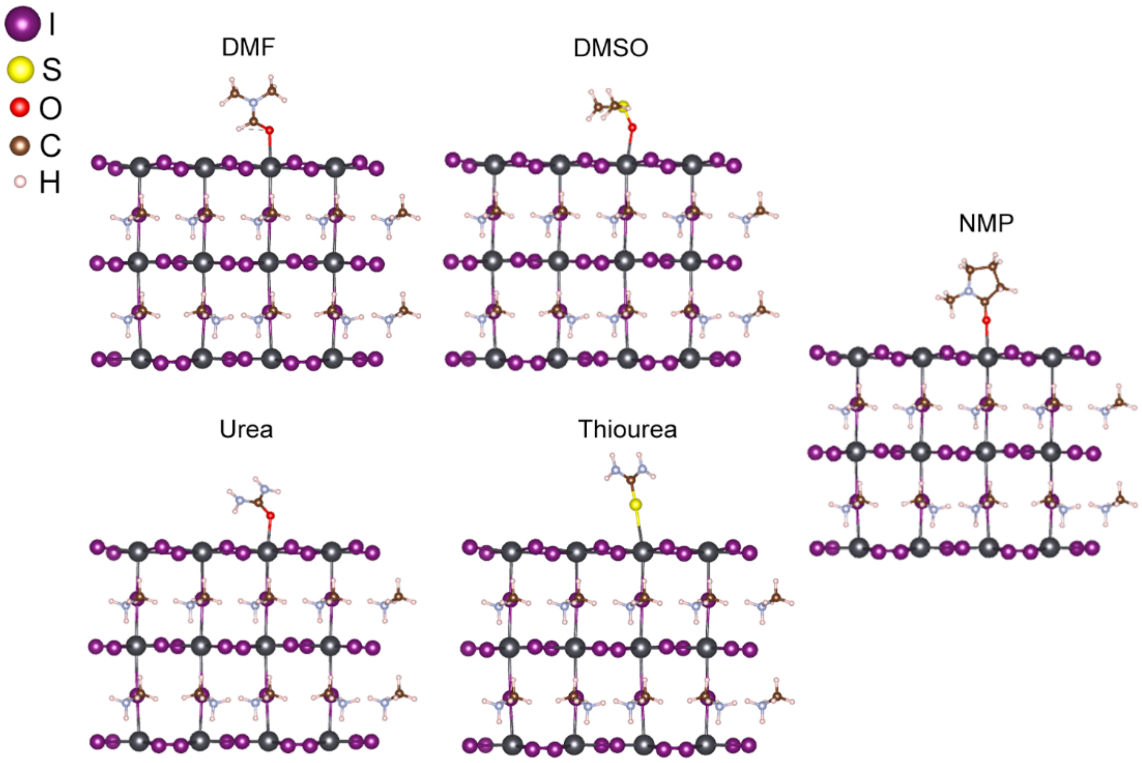

Figure S38: DMF, DMSO, NMP, urea and thiourea on the $MAPbI_3$ surface.

In conclusion, the higher polarity of thiourea enables it to form a strong bond with I^-, while its moderate adsorption energy allows it to desorb easily on the surface during annealing. These two factors make thiourea an ideal ion carrier, enhancing the mobility of I^-. Additionally, thiourea can improve the stability of $MAPbI_3$ by passivating the Pb^{2+} cations on the surface. While urea also forms a strong bond with I^-, its performance as an ion carrier is diminished due to its overly strong bond with Pb^{2+}. Therefore, as an additive, thiourea outperforms urea.

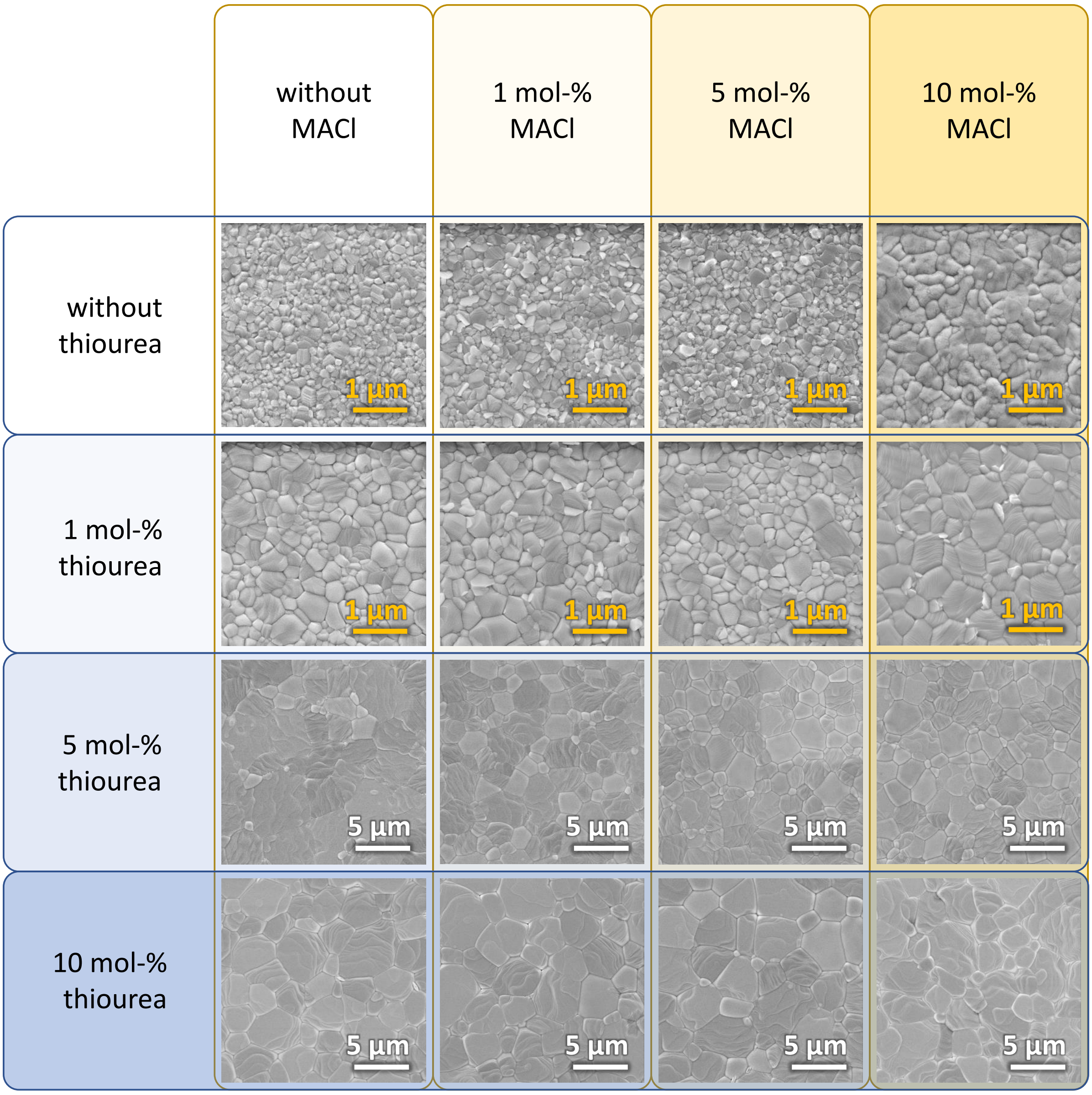

Figure S39: SEM images of $MAPbI_3$ layers employing different concentrations of both thiourea and MACl, which clearly shows that the grain morphology is largely dominated by the additive that has the strongest impact on ion mobility.

References

1. Thiesbrummel, J. *et al.* Ion-induced field screening as a dominant factor in perovskite solar cell operational stability. *Nature Energy* **9,** 664–676 (2024).
2. Cheng, F. *et al.* N-Methyl-2-pyrrolidone as an excellent coordinative additive with a wide operating range for fabricating high-quality perovskite films. *Inorganic Chemistry Frontiers* **6,** 2458–2463 (2019).
3. Zöllner, D. Grain microstructural evolution in 2D and 3D polycrystals under triple junction energy and mobility control. *Computational Materials Science* **118,** 325–337 (2016).

4. Dunlap-Shohl, W. A., Zhou, Y., Padture, N. P. & Mitzi, D. B. Synthetic Approaches for Halide Perovskite Thin Films. *Chemical Reviews* **119,** 3193–3295 (2019).

5. Scherrer, P. Bestimmung der Größe und der inneren Struktur von Kolloidteilchen mittels Röntgenstrahlen. *Nachrichten von der Gesellschaft der Wissenschaften zu Göttingen, Mathematisch-Physikalische Klasse,* 98–100 (1918).

6. Ronsin, O. J. J. & Harting, J. Phase-Field Simulations of the Morphology Formation in Evaporating Crystalline Multicomponent Films. *Advanced Theory and Simulations* **5,** 2200286 (2022).

7. Qiu, S. *et al.* In Situ Probing the Crystallization Kinetics in Gas-Quenching-Assisted Coating of Perovskite Films. *Advanced Energy Materials* **14,** 2303210 (2024).

8. Zöllner, D. Treating grain growth in thin films in three dimensions: A simulation study. *Computational Materials Science* **125,** 51–60 (2016).

9. Thompson, C. V. Grain Growth in Thin Films. *Annual Review of Materials Research* **20,** 245–268 (1990).

10. Zöllner, D. & Pantleon, W. Effect of boundary grooving on grain growth by Potts model simulations. *Journal of Physics: Conference Series* **2635,** 012033 (2023).

11. Barmak, K. *et al.* Grain growth and the puzzle of its stagnation in thin films: The curious tale of a tail and an ear. *Progress in Materials Science* **58,** 987–1055 (2013).

12. Tang, M., Reed, B. W. & Kumar, M. Coarsening kinetics of topologically highly correlated grain boundary networks. *Journal of Applied Physics* **112,** 043505 (2012).

13. Greve, C. *et al.* To Stop or to Shuttle Halides? The Role of an Ionic Liquid in Thermal Halide Mixing of Hybrid Perovskites. *ACS Energy Letters* **8,** 5041–5049 (2023).

14. Piveteau, L., Morad, V. & Kovalenko, M. V. Solid-State NMR and NQR Spectroscopy of Lead-Halide Perovskite Materials. *Journal of the American Chemical Society* **142,** 19413–19437 (2020).

15. Leupold, N. *et al.* How Methylammonium Iodide Reactant Size Affects Morphology and Defect Properties of Mechanochemically Synthesized $MAPbI_3$ Powder. *European Journal of Inorganic Chemistry* **26,** e202200736 (2023).

16. Franssen, W. M. J., van Es, S. G. D., Dervişoğlu, R., de Wijs, G. A. & Kentgens, A. P. M. Symmetry, Dynamics, and Defects in Methylammonium Lead Halide Perovskites. *The Journal of Physical Chemistry Letters* **8,** 61–66 (2017).

17. Bi, E., Song, Z., Li, C., Wu, Z. & Yan, Y. Mitigating ion migration in perovskite solar cells. *Trends in Chemistry* **3,** 575–588 (2021).

18. Blázquez, J. S., Romero, F. J., Conde, C. F. & Conde, A. A Review of Different Models Derived from Classical Kolmogorov, Johnson and Mehl, and Avrami (KJMA) Theory to Recover Physical Meaning in Solid-State Transformations. *physica status solidi (b)* **259,** 2100524 (2022).
